# Single-cell to pre-clinical evaluation of *Trem2*, *Folr2*, and *Slc7a7* as macrophage-associated biomarkers for atherosclerosis

**DOI:** 10.1093/cvr/cvaf210

**Published:** 2025-11-07

**Authors:** Tiit Örd, Senthil Palani, Judith Giroud Gerbetant, Susanna Bodoy, Tapio Lönnberg, Henri Niskanen, Aarthi Ravindran, Lari Holappa, Melody Chemaly, Mari Taipale, Kadri Õunap, Retu Haikonen, Husain Talukdar, Katyayani Sukhavasi, Heidi Liljenbäck, Jenni Virta, Anna-Kaisa Ruotsalainen, Clara Pierrot-Blanchet, Maxwell W G Miner, Olli Moisio, Noora Rajala, Xiang-Guo Li, Philip S Low, Antti Saraste, Merja Heinäniemi, Seppo Ylä-Herttuala, Johan L M Björkegren, Ulf Hedin, Ljubica Matic, Laurent Yvan-Charvet, Manuel Palacín, Anne Roivainen, Minna U Kaikkonen

**Affiliations:** A. I. Virtanen Institute for Molecular Sciences, University of Eastern Finland, P.O. Box 1627, Kuopio FI-70211, Finland; Turku PET Centre, University of Turku and Turku University Hospital, Kiinamyllynkatu 4-8, Turku FI-20520, Finland; Biosciences Department, University of Vic-Central University of Catalonia (UVic-UCC), Vic, Spain; Centro de Investigación Biomédica en Red Enfermedades Raras (CIBERER), Barcelona, Spain; Institute for Research in Biomedicine (IRB Barcelona), the Barcelona Institute of Science and Technology (BIST), Barcelona 08028, Spain; Biosciences Department, University of Vic-Central University of Catalonia (UVic-UCC), Vic, Spain; Centro de Investigación Biomédica en Red Enfermedades Raras (CIBERER), Barcelona, Spain; Institute for Research in Biomedicine (IRB Barcelona), the Barcelona Institute of Science and Technology (BIST), Barcelona 08028, Spain; Turku Bioscience Centre, University of Turku and Åbo Akademi University, Turku FI-20520, Finland; InFLAMES Research Flagship, University of Turku, Turku FI-20520, Finland; A. I. Virtanen Institute for Molecular Sciences, University of Eastern Finland, P.O. Box 1627, Kuopio FI-70211, Finland; A. I. Virtanen Institute for Molecular Sciences, University of Eastern Finland, P.O. Box 1627, Kuopio FI-70211, Finland; A. I. Virtanen Institute for Molecular Sciences, University of Eastern Finland, P.O. Box 1627, Kuopio FI-70211, Finland; Division of Vascular Surgery, Department of Molecular Medicine and Surgery, Karolinska University Hospital and Karolinska Institutet, Stockholm, Sweden; A. I. Virtanen Institute for Molecular Sciences, University of Eastern Finland, P.O. Box 1627, Kuopio FI-70211, Finland; A. I. Virtanen Institute for Molecular Sciences, University of Eastern Finland, P.O. Box 1627, Kuopio FI-70211, Finland; A. I. Virtanen Institute for Molecular Sciences, University of Eastern Finland, P.O. Box 1627, Kuopio FI-70211, Finland; Department of Medicine, Karolinska Institutet, Karolinska Universitetssjukhuset, Huddinge, Sweden; Department of Cardiac Surgery and the Heart Clinic, Tartu University Hospital and Department of Cardiology, Institute of Clinical Medicine, Tartu University, Tartu, Estonia; Turku PET Centre, University of Turku and Turku University Hospital, Kiinamyllynkatu 4-8, Turku FI-20520, Finland; Turku Center for Disease Modeling, University of Turku, Kiinamyllynkatu 10, Turku FI-20520, Finland; Turku PET Centre, University of Turku and Turku University Hospital, Kiinamyllynkatu 4-8, Turku FI-20520, Finland; A. I. Virtanen Institute for Molecular Sciences, University of Eastern Finland, P.O. Box 1627, Kuopio FI-70211, Finland; Institut National de la Santé et de la Recherche Médicale (Inserm) U1065, Université Côte d’Azur, Centre Méditerranéen de Médecine Moléculaire (C3M), Institute Hospitalo-Universitaire (IHU) RespirERA, Nice 06204, France; Turku PET Centre, University of Turku and Turku University Hospital, Kiinamyllynkatu 4-8, Turku FI-20520, Finland; Turku PET Centre, University of Turku and Turku University Hospital, Kiinamyllynkatu 4-8, Turku FI-20520, Finland; Turku PET Centre, University of Turku and Turku University Hospital, Kiinamyllynkatu 4-8, Turku FI-20520, Finland; Turku PET Centre, University of Turku and Turku University Hospital, Kiinamyllynkatu 4-8, Turku FI-20520, Finland; InFLAMES Research Flagship, University of Turku, Turku FI-20520, Finland; Department of Chemistry, University of Turku, Turku FI-20500, Finland; Department of Chemistry, Purdue University, West Lafayatte, IN, USA; Turku PET Centre, University of Turku and Turku University Hospital, Kiinamyllynkatu 4-8, Turku FI-20520, Finland; Heart Center, Turku University Hospital and University of Turku, Turku FI-20520, Finland; Institute of Biomedicine, School of Medicine, University of Eastern Finland, Kuopio, Finland; A. I. Virtanen Institute for Molecular Sciences, University of Eastern Finland, P.O. Box 1627, Kuopio FI-70211, Finland; Department of Medicine, Karolinska Institutet, Karolinska Universitetssjukhuset, Huddinge, Sweden; Clinical Gene Networks AB, Stockholm, Sweden; Division of Vascular Surgery, Department of Molecular Medicine and Surgery, Karolinska University Hospital and Karolinska Institutet, Stockholm, Sweden; Division of Vascular Surgery, Department of Molecular Medicine and Surgery, Karolinska University Hospital and Karolinska Institutet, Stockholm, Sweden; Institut National de la Santé et de la Recherche Médicale (Inserm) U1065, Université Côte d’Azur, Centre Méditerranéen de Médecine Moléculaire (C3M), Institute Hospitalo-Universitaire (IHU) RespirERA, Nice 06204, France; Centro de Investigación Biomédica en Red Enfermedades Raras (CIBERER), Barcelona, Spain; Institute for Research in Biomedicine (IRB Barcelona), the Barcelona Institute of Science and Technology (BIST), Barcelona 08028, Spain; Department of Biochemistry and Molecular Biology, Faculty of Biology, University of Barcelona, Barcelona, Spain; Turku PET Centre, University of Turku and Turku University Hospital, Kiinamyllynkatu 4-8, Turku FI-20520, Finland; InFLAMES Research Flagship, University of Turku, Turku FI-20520, Finland; Turku Center for Disease Modeling, University of Turku, Kiinamyllynkatu 10, Turku FI-20520, Finland; A. I. Virtanen Institute for Molecular Sciences, University of Eastern Finland, P.O. Box 1627, Kuopio FI-70211, Finland

**Keywords:** Atherosclerosis, Macrophages, scRNA-seq, TRAP-seq, Lipid-associated macrophages, Biomarkers, PET imaging, Glutamine metabolism, Trem2, Folr2, Slc7a7

## Abstract

**Aims:**

Atherosclerosis is a major global health challenge, with limited diagnostic and therapeutic options. Macrophages drive disease progression, but their tissue-specific phenotypes and functions remain poorly defined. This study aims to elucidate macrophage-driven mechanisms by characterizing their functional diversity across key metabolic and vascular tissues.

**Methods and results:**

We used single-cell RNA sequencing (scRNA-seq) and translating ribosome affinity purification sequencing (TRAP-seq) to profile macrophage-specific gene programmes in a mouse model of atherosclerosis across the aorta, adipose tissue, and liver. Our data highlight tissue-specific macrophage gene programmes and identify markers that are shared across mouse and human plaques. First, we identified soluble *Trem2* as a potential circulating biomarker for differentiating between asymptomatic and symptomatic individuals. Secondly, we leveraged the pronounced expression of *Folr2* and *Slc7a7* to explore the potential of folate and glutamine as positron emission tomography (PET) tracers for disease burden assessment through *in vivo* PET imaging. Finally, we show that knockout of *Slc7a7* inhibits acetylated low-density lipoprotein uptake and dampens the gene signature linked to lipid-associated macrophages. This suggests that glutamine signalling may play a critical role in foam cell formation, a key event in atherosclerosis.

**Conclusion:**

Our findings provide novel insights into macrophage-specific gene programmes during atherosclerosis progression and identify a set of promising biomarkers that can serve as a resource for future studies. These findings could significantly contribute to improving the diagnosis, monitoring, and treatment of atherosclerosis.

**Time of primary review: 112 days**

**See the editorial comment for this article ‘Lipid associated macrophages as cellular markers of cardiometabolic disease’, by T.A. Lapré and M.A. Hoeksema, https://doi.org/10.1093/cvr/cvaf263.**

## 1. Introduction

Atherosclerosis is a complex, chronic inflammatory disease characterized by lipid accumulation in the artery wall leading to plaque formation. A central player in this pathological process is the macrophage, a highly diverse and plastic cell type capable of adopting a spectrum of functional states in response to environmental cues.^1^ The concept of diversity of mouse macrophages in healthy tissue has notably been studied by the ImmGen consortium, among others,^2,3^ but it has not yet been fully explored in pathological tissues. Given their crucial role in atherosclerosis progression, a better understanding of the gene programmes defining the differentiation of macrophage subtypes across various tissues during disease progression is of the utmost importance.

Recent advances in single-cell RNA sequencing (scRNA-seq) have enabled the study of gene expression in specific cell types, including macrophages, with unprecedented resolution and across species.^1,4,5^ Much of the current knowledge, however, is derived from studies focusing on a single tissue type, such as the aorta, without observing other peripheral metabolic tissues such as liver and adipose tissue, which are known to affect plaque development through plasma metabolites as well as organ communication.^6^ Furthermore, while there has been progress in identifying macrophage-associated genes implicated in atherosclerosis, their translational potential as biomarkers for non-invasive, *in vivo* monitoring of disease burden remains largely unexplored. The development of such biomarkers could revolutionize the diagnosis and management of atherosclerosis. For instance, biomarkers tailored to subclinical diagnosis or for identifying asymptomatic patients may hold significant potential for prevention of myocardial infarction or stroke.^7,8^ Simultaneously, the advancement in novel positron emission tomography (PET) radiotracers offers the prospect of a more refined and precise characterization of high-risk plaque.^9^ Finally, the functional role of specific macrophage genes in the progression of atherosclerosis, particularly their contribution to lipid uptake and foam cell formation (a hallmark of atherosclerosis) is poorly understood. A more detailed understanding of these processes could unveil novel therapeutic targets.

In this study, we aim to address these gaps in knowledge. We employed translating ribosome affinity purification sequencing (TRAP-Seq) and scRNA-Seq to profile macrophage-specific gene programmes across multiple tissues in a mouse model of atherosclerosis. First, we identified triggering receptor expressed on myeloid cells 2 (*Trem2)* expression in vascular tissue as a marker of plaque burden and as a circulating protein partly capable of distinguishing between asymptomatic and symptomatic plaques. Next, we explored the potential of folate and glutamine as PET tracers, leveraging high expression of folate receptor (*Folr2*) and solute carrier family 7 member 7 (*Slc7a7*), respectively, in atherosclerosis-associated macrophages. Finally, we elucidated the functional role of *Slc7a7* in lipid uptake, establishing its contribution to foam cell formation. Our study offers fresh insights into macrophage gene programmes during the development of atherosclerosis and unveils potential biomarkers for monitoring disease burden.

## 2. Methods

### 2.1 Atherosclerosis disease stage course mouse experiments

To induce different stages of atherosclerosis, male mice deficient in the low-density lipoprotein receptor and expressing only apolipoprotein B100 (LDLR^−/−^ApoB^100/100^, strain #003000; Jackson Laboratory, Bar Harbor, ME, USA) were fed with a high-fat diet (HFD; 0.2% total cholesterol, 42% calories from fat, 34% sucrose by weight; TD 88137; Envigo, Madison, WI, USA). For TRAP-Seq, the LDLR^−/−^ApoB^100/100^ were crossed with mice expressing *Csf1r* promoter 3×FLAG-EGFP-RPL10a transgenic construct.^10^ The TRAP-Seq mouse strains used here have been made available in the European Mouse Mutant Archive (https://www.infrafrontier.eu/emma/)^11^ mouse repository under accession numbers EM:15591 and EM:15575.

The HFD was initiated at different ages to ensure that all groups of mice were matched for age at the end of the study. The mice fed an HFD from 5–6 months of age for 1 month were used as an early disease model, and mice fed an HFD from 3–4 months of age for 3 months were used as an advanced disease model. LDLR^−/−^ApoB^100/100^ mice fed a chow diet were used as genetic background controls (prelesion model). Finally, wild-type C57Bl/6J mice fed on a chow diet were used as unaffected controls (control group). The sample sizes for each experiment are indicated in the figure legends.

All animal experiments were approved by the national Project Authorization Board (permission numbers ESAVI/4567/2018, ESAVI/6772/2018, ESAVI/11751/2021, and ESAVI/17197/2021) and were carried out in compliance with the EU Directive 2010/EU/63 on the protection of animals used for scientific purposes.

### 2.2 TRAP-seq library generation

Tissue was frozen in liquid nitrogen, pulverized using a Cellcrusher cryo-press (Cellcrusher, Cork, Ireland) and further processed in Lysis buffer with Dounce homogenizer. Lysis buffer was freshly prepared, containing a low-salt/homogenization buffer (20 mM HEPES pH 7.4; Fisher, 10041703), 150 mM KCl (Sigma, 60142), 10 mM MgCl2 (Invitrogen, AM9530G), supplemented with 0.5 mM dithiothreitol (DTT) (Sigma, 10197777001), cOmplete Mini EDTA-free protease inhibitor (Roche 11836170001, one mini tablet/10 mL), 100 μg/mL cycloheximide (Sigma-Aldrich, 1810) in dimethylsulfoxide (Sigma C1988-1G), 0.2 U/μL murine RNase inhibitor (NEB M0314L), and 0.1 U/μL Superasin RNase inhibitor (Thermo Scientific AM2696). The buffer was added to the tissue at 1 mL per 100 mg of tissue, and homogenized samples were centrifuged at 2000 g for 10 min at 4°C to separate debris. Supernatant was collected, mixed with 10% IGEPAL CA630 (Sigma, I8896) to reach final 1%v/v and with 1,2-diheptanoyl-sn-glycero-3-phosphocholine (Avanti Polar Lipids, 850306P) in final concentration of 30 mM. Samples were incubated on ice for 5 min centrifuged at 20 000 g for 10 min at 4°C.

Biotinylated Protein L (Pierce #29997) was dissolved in phosphate-buffered saline (PBS) (1 µg/µL) and 120 µL was mixed with 300 µL of Dynabeads MyOne Streptavidin T1 (10 mg/mL; Invitrogen 65602) to form a bead–protein complex used per sample in pulldown experiments. This complex was employed to capture anti-GFP antibodies, clone 19F7 and clone 19C8, 37.5 µg each, from the Antibody and Bioresource Core Facility at Memorial Sloan Kettering Cancer Center. Beads were incubated with antibodies for 1 h in 1 mL of low-salt buffer (20 mM HEPES pH 7.4, 10 mM MgCl2, 150 mM KCl, 1% IGEPAL CA630, 0.5 mM DTT, and 100 µg/mL cycloheximide) and washed 4 times before use. To extract bulk RNA (input RNA), 5% of cell lysate was set aside and mixed with lysis buffer from the RNeasy Kit (Qiagen, 74104). The remaining lysate was incubated with pre-prepared antibody-Dynabead affinity matrix for 1–2 h at 4°C. To enhance RNA purity, the beads were then washed three times with 1 mL high-salt buffer (20 mM HEPES pH 7.4, 350 mM KCl, 5 mM MgCl2, 1% IGEPAL CA630, 1 mM DTT, and 100 µg/mL cycloheximide). RNA was extracted using the Absolutely RNA Nanoprep Kit (#400753, Agilent Technologies, Santa Clara, CA), producing the immunoprecipitated (IP) RNA sample.

RNA quality and concentration were assessed using the Bioanalyzer RNA 6000 Pico assay (Agilent). Library preparation was conducted using the SMARTer Stranded Total RNA-Seq Kit v2 (TaKaRa Bio, 634411, 634412), followed by single-end sequencing on the Illumina NextSeq 500 platform.

### 2.3 Single-cell RNA-Seq data sources and processing

scRNA-Seq from mouse aorta and epididymal white adipose tissue was obtained from the same disease stages (genotype and diet conditions) as the TRAP-Seq and PET imaging experimental groups. The generation of the aorta scRNA-Seq data (GEO accession GSE205930) is described in detail in ref. ^12^, while the adipose data (GEO accession GSE241552) is described in ref. ^13^. Briefly, mice were sacrificed, perfused with cold PBS-heparin, and dissected on ice. The extracted tissues were minced with a scalpel and enzymatically dissociated to a single-cell suspension, followed by red blood cell lysis and magnetic dead cell removal. The Chromium Single Cell 3′ Kit (v2 Chemistry; 10 × Genomics) was used for scRNA-Seq library preparation.

For mouse scRNA-Seq, data from both tissues was processed as described in our previous work.^12^ Briefly, the 10 × Genomics Cell Ranger count pipeline (version 3.0.2) with the mm10 reference package (version 3.0.0) was used for cell calling and gene quantification. Briefly, the Seurat^14^ (version 3.1.0) R package was used to first run the standard (log normalization-based) RNA processing workflow recommended by the package authors on each sample individually. DecontX^15^ (from the celda R package version 1.1.6) was used with default parameters remove ambient RNA contamination. Cells with high (>0.3) estimated contamination fraction, potentially doublets, were excluded prior to marker finding. Immune cell clusters were identified by expression of *Ptprc* (encoding CD45) and retained. Finally, immune cells across aorta and adipose libraries were integrated using the Seurat CCA method.^14^ The integrated cells were clustered using Seurat FindClusters with resolution 0.8 (default) and the resulting clusters were manually renamed after observing the top marker genes. Cell cycle phase scores were calculated using the Seurat CellCycleScoring function with the built-in gene lists for the S and G2/M phases.

To calculate marker genes in scRNA-Seq, the Seurat FindMarkers command was used to perform a Wilcoxon Rank Sum test. *P*-value <0.05 after adjustment for multiple testing by FDR was considered significant. The cutoffs used for minimum fraction of cells expressing the gene and for minimum fold change are stated in the Table headers.

Mouse liver macrophage/monocyte scRNA-Seq data from a previously published diet time course study^16^ was obtained from https://www.livercellatlas.org (access date: 7 January 2025). We used the original authors’ count matrix and cell annotations which were already subsetted to include only macrophages/monocytes. We retained all the original experimental conditions (standard and Western diet and 12-, 24-, and 36-week time points). The count matrix was processed using the default Seurat log-normalization RNA workflow (as for aorta and adipose tissue), and the 3-tissue (adipose–aorta–liver) integration of macrophage/monocyte cells was done using CCA (as for aorta–adipose integration, referenced above).

Human coronary artery scRNA-Seq was obtained from GEO (accession GSE131778) as count matrices generated by the original authors.^17^ The counts were processed using the RNA workflow suggested by the authors of Seurat version 3.1.0^14^ and cell types were annotated based on the gene lists provided in the original publication.

The protein annotations (membrane-localized, receptor, and metabolic proteins) were based on the Human Protein Atlas^18^ (release 22).

### 2.4 Biobank of Karolinska Endarterectomy (BiKE)

Patients undergoing surgery for symptomatic (S) or asymptomatic (AS), high-grade (>50% NASCET)^19^ carotid stenosis at the Department of Vascular Surgery, Karolinska University Hospital and Department of Surgery, Vascular section, Södersjukhuset, Stockholm, Sweden, were enrolled in the study and clinical data recorded on admission. Symptoms of plaque instability were defined as transitory ischaemic attack, minor stroke, and amaurosis fugax. Patients without qualifying symptoms within 6 months prior to surgery were categorized as AS and indication for carotid endarterectomy was based on results from the Asymptomatic Carotid Surgery Trial.^20^ Carotid plaques and blood samples were collected at surgery and retained within the Biobank of Karolinska Endarterectomies (BiKE). The BiKE study cohort demographics, details of sample collection, processing, and large-scale analyses were as previously described.^21–23^

For microarrays, *n* = 127 plaques were divided transversally at the most stenotic part and the proximal half of the lesion used for RNA preparation. Normal artery controls were obtained from nine macroscopically disease-free iliac arteries and one aorta from organ donors without a history of cardiovascular disease (*n* = 10).

The following five Olink^®^ panels were used in BiKE plasma profiling: Olink^®^ Target 96 Cardiometabolic, Olink^®^ Target 96 CVD II, Olink^®^ Target 96 CVD III, Olink^®^ Target 96 Development, and Olink^®^ Target 96 Immuno-Oncology. The Olink^®^ platform uses proprietary proximity extension assay (PEA) technology and a readout based on next-generation sequencing (NGS) in Illumina NovaSeq 6000. OlinkAnalyze package was used to perform the standard quality control (QC) on this panel of protein analytes using the normalized protein expression (NPX) function and relevant QC parameters as suggested by the package vignette to remove the outlier samples that do not meet their standard quality flags. A total of 100 plasma samples from BiKE patients (50 asymptomatic and 50 symptomatic) were analysed. In brief, two specific antibodies bind the target protein, bringing two single stranded DNA tags into close proximity. Then, the double stranded tag was cleaved and amplified by PCR and further indexed to allow the preparation of libraries, which were then sequenced using Illumina’s NovaSeq platform. Relative expression values ( NPX) were on a log2 scale and are derived from the qPCR step Ct values. One NPX difference corresponds to 2-fold in protein concentration.

All human samples in BiKE were collected with informed consent from patients or organ donors’ guardians. Human studies were approved by the regional Ethical Committee and followed the guidelines of the Declaration of Helsinki. The full microarray data (GSE21545) have been deposited at NCBI Gene Expression Omnibus and is publicly available. The individual human data cannot be deposited or shared because of the GDPR and ethics laws that regulate the privacy of individuals that participated in the study.

Data analysis of the selected genes of interest was done using GraphPad Prism v.10 using a two-sided Student’s *t*-test assuming non-equal deviation. Correlation analyses were performed using the Pearson method. Results are displayed as mean ± SD and threshold for significance is *P* < 0.05.

### 2.5 Stockholm-tartu atherosclerosis reverse networks engineering task (STARNET)

The STARNET study cohort demographics, details of sample collection, processing, and large-scale analyses have been previously described.^24,25^ Patients diagnosed with coronary artery disease (CAD) were enrolled at the Department of Cardiac Surgery, Tartu University Hospital. Informed consent was obtained from all subjects (Ethics Review Committee on Human Research of the University of Tartu, IRB no: 289/T-12). Each enrolled participant completed a questionnaire to assess disease history, current drug regimens, and lifestyle (e.g. daily activity, alcohol consumption, and smoking). Inclusion criteria for individuals with CAD were eligible for coronary artery bypass surgery (CABG) and, for controls, eligible for open-heart surgery for reasons other than CABG (primarily valve replacement following aortic stenosis) and a pre-operative angiogram ruling out obstructive CAD. For both individuals with CAD and controls, the absence of other severe systemic disease such as active cancer or inflammatory disease was also required. During open-breast surgery, atherosclerotic aortic arterial wall (AOR), mammary artery (MAM), only in individuals with CAD, visceral abdominal fat, subcutaneous fat, liver (LIV) and skeletal muscle (SKLM) biopsies were obtained and immediately kept in Allprotect Tissue Reagent (Qiagen) and frozen at −80°C. Preoperative blood samples were collected for biochemical screens, plasma, whole blood RNA and DNA isolations. EDTA blood tubes were centrifuged for 10 min (RT) 300 rcf (acceleration 4, deceleration 0). The plasma (supernatant) was collected in 2 mL Eppendorf tubes and frozen at −80°C.

Tissue protein isolation was done using a 10 × RIPA buffer (Sigma, R0278) with protease inhibitor cocktail (cOmplete™, Mini Protease Inhibitor Cocktail, Roche). Weigh tissue (much needed for proper addition of buffer so that protein does not get diluted). Add buffer with inhibitor in the ratio of 1:4 (i.e. to 0.1 g of tissue add 0.4 mL of buffer with inhibitor). All steps must be carried out at 2–8°C. Homogenize on ice using ultra-turrax homogeniser till the tissue is properly minced. Incubate/vortex on ice on shaker for 1 h. Centrifuge at 5000 rpm for 15 min at 4°C. Store supernatant and discard the pellet. Measure tissue protein isolated (supernatant) using Bradford reagent (ThermoFisher, Waltham, MA, USA) in microplate reader.

Proteomic profiling was conducted using the Olink Explore 3072 platform, a high-throughput PEA-based technology.^26^ Biological samples were collected and processed under standardized conditions before incubation with oligonucleotide-labelled antibody pairs. Following proximity extension, pre-amplification, and NGS on an Illumina NovaSeq 6000 system, raw sequencing data were processed using Olink’s proprietary software. NPX values were calculated using the Olink normalization algorithm to account for inter-sample variation. To assess the associations of TREM2 between plasma and tissue (AOR, LIV, MAM, SKLM) protein expression and clinical variables, Pearson correlation analysis was used.

### 2.6 PET chemicals and reagents

^68^GaCl_3_ was obtained from a ^68^Ge/^68^Ga IGG-100 generator (Eckert & Ziegler, Valencia, CA, USA) via elution with 0.1 M hydrochloric acid in water. The NOTA-folate precursor was provided by Professor Phillip Low from Purdue University, Department of Chemistry (West Lafayatte, IN, USA). The tosylated precursor for (2S,4*R*)-4-[^18^F]fluoroglutamine (^18^F-FGln) synthesis and the non-radioactive reference compound FGln were provided by the Organic Synthesis Core Facility at Memorial Sloan Kettering Cancer Center, New York, NY, USA. The cassettes for 2-deoxy-2-[^18^F]fluoro-*D*-glucose (^18^F-FDG) synthesis were purchased from GE Healthcare (Waukesha, WI, USA). Other chemicals and reagents were purchased from commercial vendors.

### 2.7 PET tracer radiosynthesis

The chemical structures and preparation of gallium-68-labelled 14,7-triazacyclononane-1,4,7-triacetic acid (NOTA)-conjugated folate (^68^Ga-FOL) and ^18^F-FGln have previously been reported.^27–29^ The preparation of ^18^F-FDG was accomplished using a fully automated cassette-based system and a FASTLab^®^ radiosynthesis device,^30^ following Good Manufacturing Practices. Prior to the release of all synthesized batches, quality control was conducted through high-performance liquid chromatography for all compounds with additional thin-layer chromatography also being used for ^18^F-FDG. ^68^Ga-FOL, ^18^F-FGln, and ^18^F-FDG have been validated for studying atherosclerosis in our previous studies.^27,28^

### 2.8 PET/CT imaging

The mice were fasted for 3–4 h prior to imaging, anaesthetized with isoflurane (4–5% induction, 1.5–2.5% maintenance), intravenously cannulated, and placed on a heating pad in the PET/CT scanner (Inveon Multimodality; Siemens Medical Solutions, Knoxville, TN, USA). The mice received ^18^F-FDG (13.9 ± 0.7 MBq), ^18^F-FGln (14.2 ± 0.8 MBq) or ^68^Ga-FOL (20.1 ± 1.3 MBq) intravenously via the tail vein cannula (Supplementary material online, *Table S13*) for a 60 min dynamic PET acquisition. An iodinated contrast agent (100 µL eXIATM160XL; Binitio Biomedical, Ottawa, ON, Canada) was intravenously injected after PET imaging, and a 10 min high-resolution CT was performed for anatomical reference. The list-mode acquired PET data was reconstructed with an iterative three-dimensional ordered subset expectation maximization using maximum a priori with shifted Poisson distribution (OSEM3D/SP-MAP) algorithm into 10 × 30 s, 5 × 60 s, 10 × 300 s time frames. CT images were reconstructed with the Feldkamp algorithm.

PET/CT images were analysed using Carimas 2.10 software (Turku PET Centre, Turku, Finland; www.turkupetcentre.fi/carimas/). Contrast-enhanced CT was used as an anatomical reference to define the regions of interest in the aortic arch, vena cava (representing blood), and myocardium, as previously described.^31^ The results of the PET/CT analysis were expressed as standardized uptake values (SUVs), taking into account the injected radioactivity dose (minus remaining dose in the cannula and tail) and animal’s body weight. To accurately quantify aortic arch uptake, the radioactivity concentration in the blood was taken into account, and the maximum target-to-background ratio (TBR) was calculated by the formula: SUV_max, aortic arch_/SUV_mean, blood_, at 40–60 min post-injection.

### 2.9 *Ex vivo* biodistribution analysis

To study the biodistribution of tracers in different tissues, mice were placed under deep isoflurane anaesthesia (4–5% induction, 1.5–2.5% maintenance). At 70 min post-injection, blood samples were obtained through cardiac puncture. After euthanizing the mice by cervical dislocation, the tissues were dissected and weighed. Radioactivity was measured using a γ-counter (Triathler 3″; Hidex, Turku, Finland). The results were expressed as SUVs calculated as radioactivity concentration (becquerels per gram of tissue) normalized for injected radioactivity dose (compensated for the remaining radioactivity in the tail and cannula) and animal body weight.

### 2.10 Autoradiography, histology, and immunostaining

Following *in vivo* PET/CT imaging and *ex vivo* gamma counting, tracer uptake in the aorta was studied in more detail using *ex vivo* digital autoradiography. The aorta was embedded in optimal cutting temperature compound, frozen on dry ice-cooled isopentane, and cut into 20 µm and 8 µm cryosections. The 20 µm cryosections were used for analysis of tracer distribution by quantitative digital autoradiography, as previously described.^31^ The sections were exposed to a Fuji Imaging Plate BAS-TR2025 (Fuji, Tokyo, Japan) for at least 4 h for ^18^F-FDG and ^18^F-FGln, and 3 h for ^68^Ga-FOL, and then scanned by a Fuji Analyzer BAS-5000. The sections were stored at −70°C after scanning, until they were stained with haematoxylin–eosin (H&E). Stained slides were scanned with a digital slide scanner (Pannoramic 250 Flash; 3DHISTECH, Ltd., Budapest, Hungary).

The analysis of autoradiographs was performed using Tina 2.1 software (Ravtest Isotopenmessgeräte, GmbH, Straubenhardt, Germany). The results were expressed as photostimulated luminescence per square millimetre (PSL/mm^2^) normalized with the injected radioactivity dose and body mass and corrected for decay of radioactivity.

Consecutive adjacent 8 µm aortic sections were used to investigate co-localization of ^18^F-FGln and ^68^Ga-FOL in Mac-3-positive macrophages, glutamine transporter Slc7a7-positive macrophages, and Folr2-positive macrophages. The sections were incubated with either anti-mouse Mac-3 antibody (working dilution 1:1000; catalogue number: 550292; BD Biosciences, Franklin Lakes, NJ, USA), anti-SLC7A7 antibody (working dilution 1:1000; catalogue number: PA5-113527; Thermo Fisher Scientific, Waltham, MA, USA), or anti-FR-β antibody (working dilution 1:150; Biorbyt, Cambridge, UK). Then, corresponding secondary antibodies were added, and a colour reaction was developed using 3.3′-diaminobenzidine (Bright-DAB, BS04-110; ImmunoLogic, Duiven, the Netherlands). Finally, slides were counterstained with haematoxylin and mounted.

Mouse hearts and livers were collected, formalin-fixed, and embedded in paraffin for histological characterization of atherosclerotic lesions at the level of the aortic root and hepatic steatosis and steatohepatitis.^28,31^ Aortic sections of 6 µm thickness were cut transversely at the level of the coronary ostia, and consecutive sections were stained with H&E, modified Movat’s pentachrome^31,32^ and anti-Mac-3 (1:100; product: as above), anti-iNOS (1:100, catalogue number: ab15323; Abcam, Cambridge, UK), anti-MRC-1 (1:2000, catalogue number: ab64693; Abcam, Cambridge, UK), anti-SLC7A7 (1:1500; product: as above), anti-FR-β (1:150; product: as above), and anti-TREM2 (1:2000, catalogue number: PA5-87933; Thermo Fisher Scientific Waltham, MA, USA) antibodies followed by subsequent second-stage antibodies and colour reaction developed using DAB. Liver sections were stained with H&E. The stained slides were scanned with a slide scanner (Pannoramic Flash) and the images were captured using CaseViewer 2.4 software (3DHISTECH Ltd., Budapest, Hungary).

### 2.11 Quantification of soluble TREM2 (sTREM2) protein from mouse plasma

Mouse blood was collected into an EDTA Microtainer collection tube (BD Medical) and centrifuged to obtain plasma. For mouse sTREM2 ELISA assay, a 96-well plate was coated with anti-TREM2 antibody (MAB17291-100, 1:1000, R&D Systems) in coating buffer (50 mM bicarbonate buffer, pH 9.6) and incubated overnight at 4°C. After incubation, the plate was washed three times with washing buffer (0.05% Tween-20 in PBS). Next, the plate was blocked with blocking buffer (1% Bio-Rad Block Ace in PBS) for 4 h at RT, washed with PBS, and then incubated with 15 µL of plasma samples and 85 µL of assay buffer (1% bovine serum albumin and 0.05% Tween-20 in PBS) overnight at 4°C. Recombinant mouse TREM2 (50149-M08H, Sino Biological) was employed to create standard curves. The plates were washed five times with washing buffer, followed by a 1-h incubation at RT with a biotinylated mouse anti-TREM2 antibody (BAF1729, 1:3000, R&D Systems). After three more washing steps, the plates were treated with Streptavidin Poly-HRP40 Conjugate (65R-S104PHRP, 1:3000, Fitzgerald) for 1 h in the dark. After five additional washes with washing buffer, the plates were developed by adding the TMB substrate (3,3′,5,5′-Tetramethylbenzidine Liquid Substrate, Super Slow, T5569, Sigma-Aldrich). The reaction was terminated by adding stop solution (1M H_3_PO_4_) and the samples were read at 450 nm using a CLARIOstar microplate reader (BMG Labtech).

### 2.12 Primary bone marrow-derived macrophages from *Slc7a7* knockout mouse model

*Slc7a7*-LysM mice^33,34^ were generated in C57Bl/6J genetic background and all experiments were conducted under the approved project (DARP n°9177) by Use Committee from Parc Científic from Barcelona.

Bone marrow (BM) cells were obtained from 12-week-old mice. Mice were euthanized by placing them in a CO_2_ chamber for approximately 20 s followed by cervical dislocation. No anaesthesia was administered to the mice. After sacrificing, the femurs and tibiae were extracted, and BM cells were flushed out. The cell suspension was lysed for 5 min in erythrolysis buffer (R&D Systems, Minnesota, USA) at room temperature and then washed, resuspended, and cultured in 3 different 15-cm diameter plates, for 7 days, with Dulbecco's modified eagle medium supplemented with 10% heat-inactivated foetal bovine serum, 50 U/mL penicillin, 50 μg/mL streptomycin, and 50 ng/mL of recombinant macrophage colony-stimulating factor (Peprotech, Massachusetts, USA) or 30% of L-Cell conditioned medium. Six days after seeding, cells were harvested and re-seeded with the previously mentioned conditioned medium for 24 h. For foamy macrophage experiments 30 000 cells were seeded, and macrophages were treated for 24 h with either 100 μg/mL oxidized-LDL, 50 μg/mL acetylated-LDL or vehicle (PBS). Cells were then washed and stained with Oil Red O staining to measure lipid droplets inside macrophages. For RNA-Seq analysis, 100 000 cells were seeded and treated with either 100 μg/mL oxidized-LDL, 50 μg/mL acetylated-LDL or vehicle (PBS) for 24 h. Total RNA was isolated using the Qiagen RNeasy Micro Plus kit with the QIAshredder option for lysate homogenization and the gDNA eliminator column for removal of genomic DNA. RNA-Seq libraries were prepared using the SMARTer Stranded Total RNA-Seq Kit v2 (TaKaRa Bio) according to the manufacturer’s recommendations and the libraries were single-end sequenced on an Illumina NextSeq 500 instrument.

### 2.13 Bone marrow transplantation and [^14^C]glutamine uptake

Animal protocols were approved by the Institutional Animal Care and Use Committee of the French Ministry of Higher Education and Research and the Mediterranean Center of Molecular Medicine (Inserm U1065) and were undertaken in accordance with the European Guidelines for Care and Use of Experimental Animals.

Female *Ldlr^−/−^* recipient mice were irradiated 16 h prior to BM transplantation and then received an *i.v.* injection of 4 × 10^6^ BM cells extracted from *Slc7a7*-LysM knockout^34^ donor mice. The mice were allowed to recover for 5 weeks before being placed on an atherogenic diet (Western diet, TD88137, Ssniff) for 11 weeks. ^14^C-labelled glutamine uptake was assessed as described previously.^35^ A total of 2 μCi of ^14^C-labelled glutamine was *i.v.* injected, and the mice were euthanized 15 min later. Aortas were collected, and the surrounding adipose tissue was carefully dissected and removed. The aortic tissues were homogenized in a 5% HClO_4_ solution, and the radioactivity in the extract was measured and expressed as total radioactivity per sample.

### 2.14 RNA-Seq and TRAP-seq data processing

For both RNA-Seq and TRAP-Seq, sequencing reads were processed using the nf-core^36^ RNA-Seq pipeline to carry out adapter and quality trimming using Trim Galore (https://doi.org/10.5281/zenodo.7598955) followed by alignment to the mm10 mouse genome with STAR^37^ and gene quantification using the Ensembl release 93 mouse transcriptome.^38^ Lowly expressed genes were filtered out using the edgeR^39^ (version 3.24.3) function filterByExpr, requiring at least 15 counts per group and 5 counts in at least one sample in the case of TRAP-Seq. For BMDM RNA-Seq, the requirements were 10 and 2 counts, respectively, on account of their lower sequencing depth. DESeq2^40^ (version 1.22.2) was used for differential expression testing and *P* < 0.05 after adjustment for multiple testing by FDR was considered significant. To define macrophage-enriched genes in TRAP-Seq, DESeq2 was used to compare the counts from the macrophage-enriched RNA samples (IP fraction) to the unenriched RNA samples (INPUT fraction) from the same tissue samples. Genes with FDR < 0.05 and log2 fold change >1 were considered macrophage-enriched. The fgsea R package^41^ (version 1.25.1) was used to perform gene set enrichment analysis. Reactome pathways were obtained from MSigDB (mouse release 2024.1).^42^ All pathways with 10–1000 genes were considered. To filter significantly enriched pathways into a set of non-redundant pathways, the collapsePathways function from the fgsea package was run with default parameters.

### 2.15 Statistical analysis

Results are presented as the mean ± standard deviation (SD). A two-tailed unpaired Student’s *t* test in Microsoft Excel was used to analyse differences between the groups. *P*-values <0.05 were considered statistically significant.

## 3. Results

### 3.1 Identification of cross-tissue macrophage markers for disease monitoring using TRAP-seq

**Figure 1:**
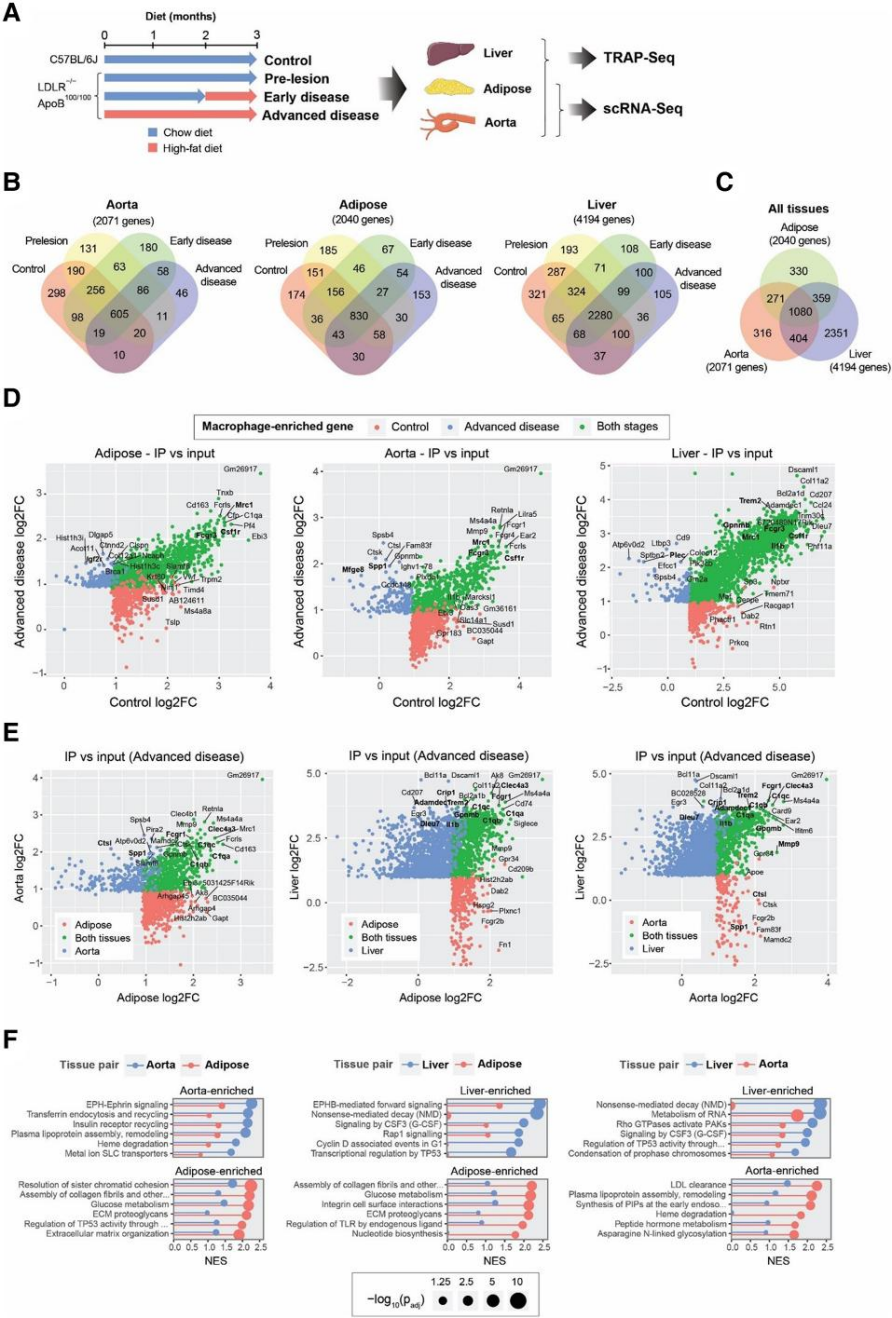
Identification of macrophage specific gene expression using TRAP-seq. (*A*) Schematic overview of the experimental setup. (*B*) Venn diagrams comparing the number of genes displaying macrophage-enriched expression in TRAP-Seq of aorta, adipose tissue, and liver, comparing between control, prelesion, early and advanced disease stages. Macrophage-enriched expression was defined as FDR < 0.05 and log2 fold change (log2FC) > 1 between the TRAP-Seq immunoprecipitated RNA and the input RNA. (*C*) Venn diagram compiling the macrophage-enriched genes in adipose, aorta and liver tissues across all disease stages, illustrating the common and unique macrophage-specific gene expression. Macrophage-enriched expression was defined as in panel *B*. (*D*) Scatter plots comparing the gene expression changes between control (chow diet) and advanced disease stage (3 months high-fat diet [HFD]) in adipose tissue (left), aorta (middle), and liver (right). (*E*) Scatter plots comparing the macrophage-enriched gene expression between adipose tissue, aorta, and liver of LDLR^−/−^ApoB^100/100^ mice fed a HFD for 3 months (indicative of advanced disease stage). For panels *D* and *E*, each scatter plot shows the log2 FC of TRAP IP vs. input RNA, comparing between either two disease stages (*D*) or tissues (*E*). Blue and red dots indicate genes classified as markers in one comparison, and green indicates marker in both conditions. (*F*) Gene set enrichment analysis of Reactome pathways in TRAP-Seq data from three tissues. In each tissue, significantly enriched pathways were selected and filtered to remove redundant pathways. Comparing two tissues, the pathways with the largest differences in enrichment (normalized enrichment score) were plotted. *P*_adj_, enrichment *P*-value adjusted by FDR. For panels *B*‐*F* (TRAP-Seq), six mice per group were analysed.

Cross-tissue atlases provide insights into the tissue-specific and tissue-agnostic attributes of cell types playing roles in disease. Here, we sought to investigate the macrophage-specific gene expression profiles across atherosclerosis-relevant tissues using the LDLR^−/−^ApoB^100/100^ mouse model subjected to 0 (prelesion), 1 (early disease), and 3 (advanced disease) months of HFD, alongside C57Bl/6J control mice (*Figure 1A*). Upon examination of aortic root sections using histology, we observed atherosclerotic lesions with increasing intima-to-media ratio as the disease progressed (see Supplementary material online, *Figure S1A* and *B*). In the advanced stage, lesions exhibited a prominent necrotic core and a fibrous cap (see Supplementary material online, *Figure S1A*). As a result of HFD feeding, body weight increased by 27.6% and 32.1%, for 1- and 3-month HFD, respectively, compared to chow diet-fed mice (see Supplementary material online, *Figure S1C*). In the liver, micro- and macrovesicular steatosis was evident after 1 or 3 months of HFD, along with mild infiltration and activation of immune cells, but the protocols did not induce steatohepatitis (see Supplementary material online, *Figure S1D*).

First, we utilized the TRAP-Seq technique to characterize the macrophage-specific translatome within the aorta, liver, and adipose tissue of the mice. TRAP-Seq is a cost-effective technique that allows the selective isolation of translated mRNAs from specific cell types, in this case, macrophages, by driving the expression of EGFP-RPL10a fusion gene under the *Csf1r* promoter^10^ (see Supplementary material online, *Figure S2A* and *B*). The macrophage specific tagging was confirmed by histological analysis of the EGFP-RPL10a transgene expression (see Supplementary material online, *Figure S2C* and *D*), as well as the enrichment of macrophage markers *Csfr1*, *Lyz2* (encodes LysM), *Adgre1* (F4/80), *Lamp2* (Mac-3), *Nos2* (iNOS) and *Mrc1*, and the depletion of tissue-specific markers *Apoa1* (liver; hepatocyte), *Myl9* (aorta; smooth muscle cell), and *Lep* (adipose tissue; adipocyte) (see Supplementary material online, *Figure S3*). To assess the tissue-specific and temporal enrichment of macrophage-specific genes, we analysed the differences between the immunoprecipitated (IP) RNAs and the input RNA. This analysis identified a substantial number of macrophage-enriched genes (defined as FDR < 0.05 and log_2_ fold change >1), including 2071genes in the aorta, 2040 genes in adipose tissue, and 4194genes in the liver (*Figure 1B* and *C*, Supplementary material online, *Tables S1*–*S3*). The increased number of results in liver appears to be a consequence of larger effect sizes (IP vs. Input ratios) as well as lower variability between replicates, particularly for IP samples (see Supplementary material online, *Figure S4A* and *B*). Total sequencing depth and the number of genes detected were similar for all tissues (see Supplementary material online, *Figure S4C* and *D*). The bulk tissue (Input) expression levels of *Csf1r* were also similar (see Supplementary material online, *Figure S3*). Since the lower variability was especially evident among liver IP samples (see Supplementary material online, *Figure S4B*), it is possible that the overall high RNA content of liver (per mg of tissue) led to a more efficient TRAP process.

Overall, the contributions of tissue and disease appeared to be of the same order of magnitude for each tissue, with a couple hundred genes being associated mostly with tissue or mostly with disease stage. A large fraction of disease stage associated genes identified through TRAP-Seq were shared across the three tissues as exemplified by *Csf1r*, *Mrc1*, and *Fcgr3* (*Figure 1C* and *D*, Supplementary material online, *Tables S1*–*S3*). Still, several disease stage-specific genes were identified including significant enrichment of *Spp1* and *Mfge8* in the aorta from prelesion stage onwards and a gradual increase of *Igf2r* in adipose tissue and *Plec* in liver during disease progression.

We also saw that hundreds of genes consistently showed enrichment across tissues, supporting the similarity of macrophage gene expression programmes and the possibility to identify changes which are associated with systemic inflammatory effects of atherosclerotic conditions (*Figure 1E*, Supplementary material online, *Tables S1*–*S3*). The top genes include *Mmp9*, *Clec4a3*, *Fcgr1*, and members of the *C1q* family (*C1qa-c*). Again, also tissue-specific genes were identified, as demonstrated by liver-specific expression of *Adamdec1*, *Dleu7*, and *Crip1*, markers of Kupffer cells, and aorta-specific expression of *Ctsl* and *Spp1*.

Pathway enrichment analysis (see Supplementary material online, *Table S4*) revealed that general immune and macrophage gene sets were the most strongly over-represented across all three TRAP-Seq tissues, consistent with macrophage expression profiles. Comparative enrichment analysis between tissues also highlighted distinct features, including lipoprotein and haeme processing in the aorta and extracellular matrix-related functions in adipose tissue (*Figure 1F*). Taken together, these findings suggest that while there is a core set of macrophage genes across different tissues, there are also unique sets of genes that are specifically regulated in a tissue-dependent manner. This supports the evidence that macrophages adapt their gene expression in response to the local environment but also demonstrates disease stage-specific differences. Identifying these genes could provide a valuable resource for further investigation.

### 3.2 Monitoring the macrophage subset dynamics at single cell level

**Figure 2:**
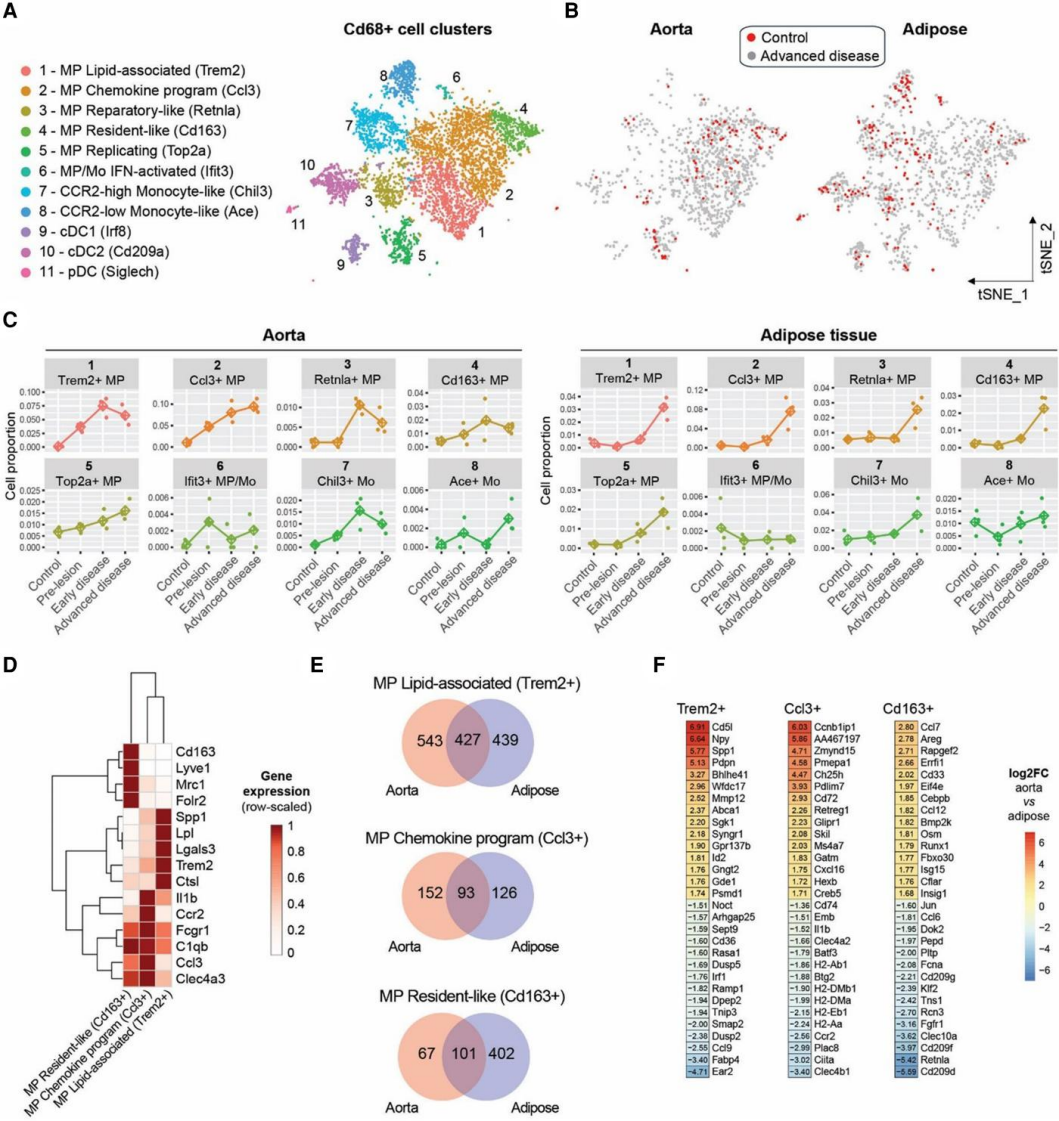
Clustering and monitoring of macrophage and monocyte changes during atherosclerosis progression in aorta and adipose tissue using scRNA-Seq. (*A*) tSNE projection of the scRNA-Seq profiles of Cd68+ myeloid cells represented as eleven manually annotated clusters from combined analysis of aorta and adipose tissue. (*B*) tSNE plot of Cd68+ myeloid cells specifically in the aorta and adipose tissue in control and advanced disease mice. (*C*) Relative changes in the cell state proportions during different stages of atherosclerosis shown for each of the three biological replicates in aorta and adipose tissue. (*D*) Gene expression of selected genes in the three most abundant macrophage clusters identified across aorta and adipose tissue. Pseudobulk gene expression (counts per million; CPM) was calculated per cluster across mouse aorta and adipose tissue scRNA-Seq cells. Gene expression is shown scaled per gene from 0 to 1 (maximum observed). (*E*) Number of marker genes detected as cluster markers in one or both tissues for the three most abundant macrophage subtypes shared between aorta and adipose tissue. (*F*) The most differential genes between aorta and adipose tissue amongst macrophages of the same cluster (markers from *E*). For the three most abundant macrophage clusters, the top tissue-specific genes are shown based on log2 fold change (log2FC) derived from pseudobulk gene expression analysis. Highly expressed genes were selected (at least 100 CPM in the higher expressing tissue). Positive log2FC denotes higher expression in aorta. For all panels, 3 mice per group were analysed.

While TRAP-Seq provides valuable insights into gene expression patterns, it possesses inherent limitations in discerning variations between individual cellular subtypes. Specifically, alterations observed in bulk RNA-Seq data, like that from TRAP-Seq, can arise from either shifts in cell subtype proportions or genuine differential gene expression within a cell type. To circumvent this ambiguity and achieve a more granular understanding of cell subtype-specific changes, we employed scRNA-Seq on the aorta and adipose tissue using the same mouse model (*Figure 1A*). Immune cells were investigated from scRNA-Seq datasets generated using the 10 × Genomics Chromium platform. Our analytical approach integrated data from both tissues to identify both common and tissue-specific subtypes, focusing on CD68 as a pan-myeloid marker. *Cd68*-expressing cells were selected based on their unique lineage marker gene expression profiles, distinguishing them from other immune cells (see Supplementary material online, *Figures S5A* and *B* and *S6A*; Supplementary material online, *Table S5*). Our analysis identified 11 Cd68+ myeloid clusters, which were named based on one of a top marker gene (*Figure 2A*; Supplementary material online, *Figure S6B*; Supplementary material online, *Table S6*). This included six macrophage subtypes (*Trem2*+ lipid-associated macrophages [LAM], *Ccl3*+ proinflammatory, Retnla + reparatory-like, *Cd163*+ resident, *Top2a* + replicating, and *Ifit3+* interferon (IFN)-activated macrophages), two monocyte subtypes (*Chil3*+ Ccr2-high and *Ace* + Ccr2-low monocytes), and three dendritic cell subtypes (*Ifit8*+, *Cd209a*+, and *Siglech* + dendritic cells) (*Figure 2A*). Adipose tissue displayed a wider myeloid cell spectrum than aorta (*Figure 2B*), and atherosclerosis increased the number of cells in all macrophage and monocyte clusters (*Figure 2C*) and several other immune cell types (see Supplementary material online, *Figure S7A* and *B*) in both tissues. Interestingly, while these subtypes illustrated a relatively gradual rise in the aorta, the adipose tissue marked a more distinct surge only during the advanced disease phase, highlighting temporal variations between tissues.

To evaluate the effect of tissue within the same transcriptomic cluster of cells, we concentrated on the three major macrophage clusters identified in both aorta and adipose tissues: Trem2+ (lipid-associated), Ccl3+ (chemokine programme), and Cd163+ (resident-like) macrophages (clusters 1, 2, and 4 in *Figure 2A*, with characteristic gene expression markers shown in *Figure 2D*). The results revealed that while many marker genes were shared between the tissues, a considerable amount were only detected in one tissue (*Figure 2E*; Supplementary material online, *Tables S7* and *S8*). For some of the tissue-specific marker genes, the expression difference between aorta and adipose macrophages within the same transcriptomic cluster was substantial (*Figure 2F*). Notably, top tissue-differential genes pointed towards different types of lipids in aorta and adipose: cholesterol in aorta (*Abca1*, *Ch25h*) and fatty acid in adipose (*Fabp4*). Thus, marker variation within macrophage clusters may indicate distinct functional specialization, as has been described for resident macrophages in tissue homeostasis.^43^

Given that our scRNA-Seq experiment lacked liver data, we additionally performed macrophage/monocyte scRNA-Seq data integration with a previously published liver dataset where HFD was fed for up to 36 weeks.^16^ The original authors classified monocytes and macrophages into 9 subtypes (see Supplementary material online, *Figure S8A*), and HFD duration considerably increased the fraction of LAMs, monocyte-derived Kupffer cells (moKCs) and an intermediate ‘mac1’ population (see Supplementary material online, *Figure S8B*). Integration of monocytes and macrophages from aorta, adipose tissue and liver resulted in nine clusters (see Supplementary material online, *Figure S8C*) and overlaying the original cell annotations revealed that cells are clustering by subtype rather than by tissue of origin (see Supplementary material online, *Figure S8D*). In the post-integration clusters, LAMs from all three tissues mostly grouped together (cluster 4), as did resident-like macrophages and Kupffer cells (cluster 1), proliferating macrophages (cluster 6), and two types of monocytes (clusters 5 and 2) (see Supplementary material online, *Figure S8E*). Chemokine and reparatory macrophages from adipose/aorta grouped with liver mac1, capsule macrophages and transitioning monocytes (cluster 0; Supplementary material online, *Figure S8E*). For each of the three tissues, three clusters (0, 1, and 4) accounted for the majority (79–91%) of macrophages (see Supplementary material online, *Figure S8F*) and resembled the chemokine/inflammatory, resident-like, and lipid-associated subtypes, respectively (see Supplementary material online, *Figure S8G*), similarly to the subdivision found in adipose and aorta alone (*Figure 2D* and *E*). Using marker genes for each integrated cluster in each tissue (see Supplementary material online, *Figure S8H*, Supplementary material online, *Table S9*), pathway enrichment analysis revealed that the transcriptomically defined cell clusters tend to have similar enrichment profiles irrespective of tissue, supporting functional convergence across tissues (see Supplementary material online, *Figure S8I*, Supplementary material online, *Table S10*). Finally, visualizing the common cluster markers (see Supplementary material online, *Figure S8H*) as gene set expression scores in the adipose–aorta–liver integrated UMAP highlights that these cluster gene programmes exist as a continuum of activation levels, with areas of overlap (see Supplementary material online, *Figure S8J*). The importance of mixtures of programmes was recently illustrated by the discovery of ‘inflammatory LAMs’ in human (but not mouse) plaques, with LAMs of other tissues remaining to be explored.^8^

In aorta and adipose tissue scRNA-Seq, and in the external liver data,^16^ proliferating macrophages represented the fourth most abundant macrophage cluster (see Supplementary material online, *Figure S8F*) and appeared distinct from the rest of the macrophages (see Supplementary material online, *Figure S8C*). In the integrated dataset of aorta, adipose and liver macrophages, cell cycle phase scoring as well as individual cell cycle marker genes confirmed the clustering of cycling cells into a distinct group (see Supplementary material online, *Figure S9A* and *B*). Focusing on this cluster revealed a divergence into branches characterized by either LAM or resident-like macrophage markers (see Supplementary material online, *Figure S9C*), highlighting that while the replicating macrophage cluster is defined by cell cycle genes, the cells also express markers of non-cycling macrophage subtypes. By tissue, macrophages from adipose and aorta expressed LAM genes, whereas cycling macrophages in the liver expressed either LAM or resident markers (see Supplementary material online, Figure  *S9D*), suggesting that multiple macrophage subtypes undergo cell cycling in the liver, consistent with previous observations.^16^

### 3.3 Identification of Trem2 as a circulating protein partly distinguishing between asymptomatic and symptomatic plaques

**Figure 3:**
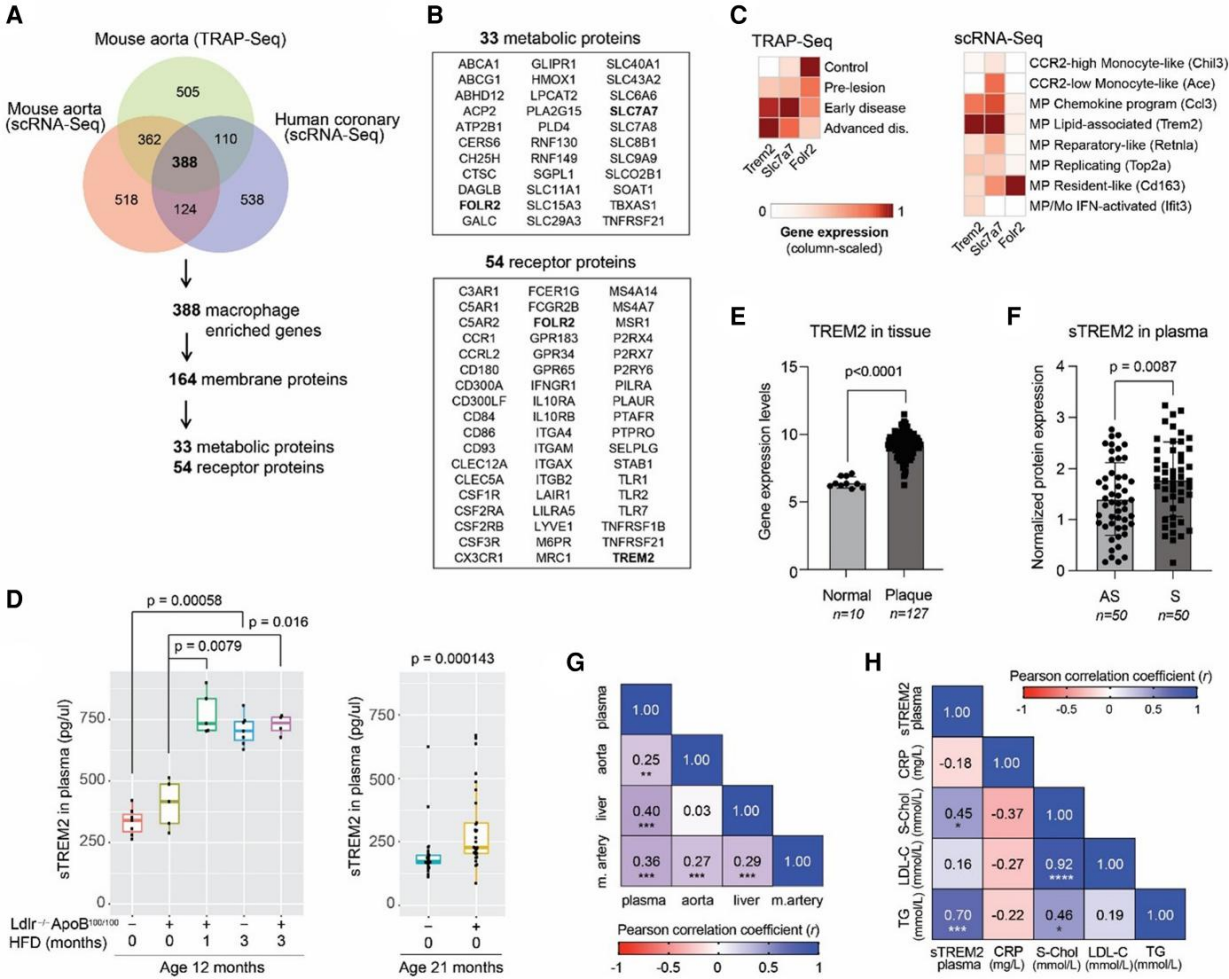
Comparative analysis of gene expression and sTREM2 levels in mouse and human samples. (*A*) Venn diagram illustrating the overlap of gene expression profiling results from mouse aorta samples analysed by TRAP-Seq and scRNA-Seq, and human coronary arteries analysed by scRNA-Seq, revealing a subset of genes enriched in macrophages. The analysis identifies 388 macrophage-enriched genes, of which 164 are membrane proteins, 33 are metabolic proteins, and 54 are receptor proteins. Sample sizes: TRAP-Seq *n* = 6 mice, mouse scRNA-Seq *n* = 3 mice, and human scRNA-Seq n = 4 patients. (*B*) Detailed lists of identified proteins categorized into metabolic proteins and receptor proteins from *A*. (*C*) Expression of *Trem2*, *Slc7a7* and *Folr2* in TRAP-Seq and scRNA-Seq of mouse aorta. For TRAP-Seq, values represent the average transcripts per million across IP replicates, while for scRNA-Seq, they correspond to cluster pseudobulk counts per million. Gene expression for each method is scaled per gene from 0 to 1, with 1 representing the maximum observed expression. Sample sizes are as indicated in panel *A*. (*D*) Quantification of soluble (s)TREM2 in the plasma of mice carrying (+) or not carrying (−) the hypercholesterolemic LDLR^−/−^ApoB^100/100^ mutations. The mice were subjected to either a regular diet (0) or a high-fat diet (HFD) for 1 or 3 months. Data are presented for both 12-month-old and 21-month-old mice, illustrating the impact of diet and age. The Wilcoxon rank sum test was used for comparing groups. Sample size (mice per group) at 12 months was 7 (−; HFD 0), 5 (+; HFD 0), 5 (+; HFD 1), 7 (−; HFD 3) and 4 (+; HFD 3), and at 21 months was 25 (−) and 30 (+). (*E*, *F*) Normalized protein expression (NPX levels of TREM2 in (*E*) human atherosclerotic tissue and (*F*) plasma from individuals of the BiKE cohort in individuals with asymptomatic (AS) and symptomatic (S) carotid stenosis. NPX was calculated as recommended by Olink and is on a log_2_ scale in arbitrary units (one NPX difference corresponds to 2-fold in protein concentration). Each dot represents an individual patient's sTREM2 levels, with 50 patients in each group. Statistical significance was measured using a two-sided Student’s *t*-test with unequal variance. (*G*) Heatmap of Pearson correlation coefficients between plasma and tissue TREM2 levels (atherosclerotic aortic arterial wall = aorta, liver, and mammary artery = m.artery) in 165 coronary artery disease patients from the STARNET cohort. (*H*) Pearson correlation matrix showing relationships between plasma sTREM2, C-reactive protein, serum cholesterol, low-density lipoprotein cholesterol, and triglycerides in 20 individuals from the BiKE cohort. For both panels, the colour scale represents the Pearson correlation coefficient (*r*), ranging from −1 to +1. Asterisks denote statistical significance: *P < 0.05, **P < 0.01, ***P < 0.001, ****P < 0.0001.

Next, we integrated the marker gene data from both TRAP-Seq and scRNA-Seq, with macrophage markers from human plaque scRNA-Seq,^17^ to pinpoint universal markers for disease-associated macrophages (see Supplementary material online, *Table S11*). Altogether, our analysis identified 388 macrophage-enriched genes (*Figure 3A*) of which 70% (274/388) were further supported as macrophage-specific in the Human Protein Atlas body-wide scRNA-Seq atlas^44^ (see Supplementary material online, *Table S12*). This list thus represents a comprehensive set of candidate genes for further exploration in macrophage biology and potential therapeutic intervention and diagnostic applications in atherosclerosis and related diseases. We subsequently directed our attention to membrane-bound receptor or metabolic proteins, given their potential as accessible diagnostic markers, facilitating cell-type-specific non-invasive disease detection and monitoring. Altogether 164 membrane proteins were identified, of which 54 represented receptors and 33 metabolic proteins (*Figure 3A* and *B*; Supplementary material online, *Table S12*).

Among the receptor proteins, we identified *Trem2*, specifically associated with LAMs and exhibiting increased gene expression in atherosclerotic tissue (*Figure 3C*; Supplementary material online, *Figures S10* and *S11A* and *B*). *Trem2*+ LAMs have been previously observed in healthy human tissues as well as in pathological settings, including atherosclerotic aorta,^1,45^ heart failure,^46^ adipose and liver tissue during obesity,^47^ damaged and fibrotic liver,^48–50^ and lung.^51^ Recent studies have demonstrated that circulating soluble (s)TREM2 can act as an indicator of *Trem2*+ macrophage recruitment to fibrotic tissues in mouse models of metabolic dysfunction-associated fatty liver disease.^52,53^ In human patients, sTREM2 more accurately differentiated between the absence or presence of mild metabolic dysfunction-associated steatohepatitis (MASH) and its advanced stages than conventional liver disease-associated markers. Also, serum sTREM2 has been recently suggested to predict cardiovascular death.^54^ Prompted by these findings, we sought to investigate whether sTREM2 could similarly function as a potential biomarker for atherosclerosis and potentially offer a means of distinguishing between patient subgroups, thereby facilitating patient stratification. Our results showed a robust and comparable increase in systemic sTREM2 levels in both LDLR^−/−^ApoB^100/100^ and wild-type mice upon HFD feeding (*Figure 3D*, left panel), confirming the prominent role of diet on sTREM2 levels.^52^ Additionally, there was a mild but statistically significant increase in sTREM2 levels in LDLR^−/−^ApoB^100/100^ mice compared to wild type when both were fed a chow diet (*Figure 3D*, right panel).

A recent study from the Carotid Artery Risk for Atherosclerosis Study reported an association between elevated sTREM2 levels and carotid plaque progression.^55^ To further investigate the underlying mechanisms and assess tissue-level expression of TREM2 in advanced human atherosclerosis, we evaluated its association with plaque burden in patients undergoing surgery for high-grade carotid stenosis and CAD. For this, we leveraged transcriptomic and proteomic data from two independent cohorts: the Biobank of Karolinska Endarterectomies (BiKE)^21–23^ and the Stockholm-Tartu Atherosclerosis Reverse Network Engineering Task (STARNET).^24,25^ In line with *TREM2* gene expression in lesional macrophages, we observed significantly elevated TREM2 protein expression in carotid plaques compared to normal arteries (*Figure 3E*). Notably, our study extends previous findings^55^ by showing that sTREM2 levels are significantly higher in symptomatic patients compared to asymptomatic individuals within the same cohort (*Figure 3F*). This suggests that beyond its expression in tissue, sTREM2 may serve as a clinically relevant biomarker of plaque instability and disease severity.

To further explore the systemic sources and correlates of circulating sTREM2, we assessed its relationship with tissue-level TREM2 expression in paired samples from early-stage lesions (mammary artery), advanced atherosclerosis (aorta), and liver. Plasma sTREM2 levels correlated significantly with TREM2 expression in all three tissues, with the strongest correlation observed in the liver (*Figure 3G*). Supporting a metabolic link, plasma sTREM2 levels positively correlated with lipid parameters including total cholesterol, LDL, HDL, and triglycerides, but not with C-reactive protein, a systemic inflammation marker (*Figure 3H*; Supplementary material online, *Figure S12*). Together, these findings suggest that sTREM2 may reflect tissue-specific TREM2 protein level and associate it with lipid metabolism and plaque phenotype.

### 3.4 Exploiting folate and glutamine transport to trace atherosclerosis progression using PET

We next sought to identify potential biomarkers that could act as PET tracers, allowing for non-invasive imaging and real-time monitoring of disease progression in the spatial context. We identified folate receptor beta (*Folr2*; *FR-β*) and solute carrier family 7 member 7 (*Slc7a7),* which are especially promising receptors/transporters because their substrates, folate (FOL) and glutamine (Gln), respectively, can be readily radiolabelled for PET investigation. *Slc7a7* expression was most strongly associated with LAMs, while *Folr2* represents a marker of resident macrophages (*Figure 3C*). The macrophage-specific expression of these genes was confirmed via immunohistochemical staining of the aortic root sections of atherosclerotic mice (see Supplementary material online, *Figure S10*) and scRNA-Seq of human and mouse atherosclerosis (see Supplementary material online, *Figure S11A* and *B*). The expression levels of *SLC7A7* and *FOLR2* were significantly elevated in human atherosclerotic plaques compared to normal tissue, and were also mildly but significantly increased in symptomatic patients vs. asymptomatic patients within the BiKE cohort (see Supplementary material online, *Figure S13A* and *B*).

**Figure 4:**
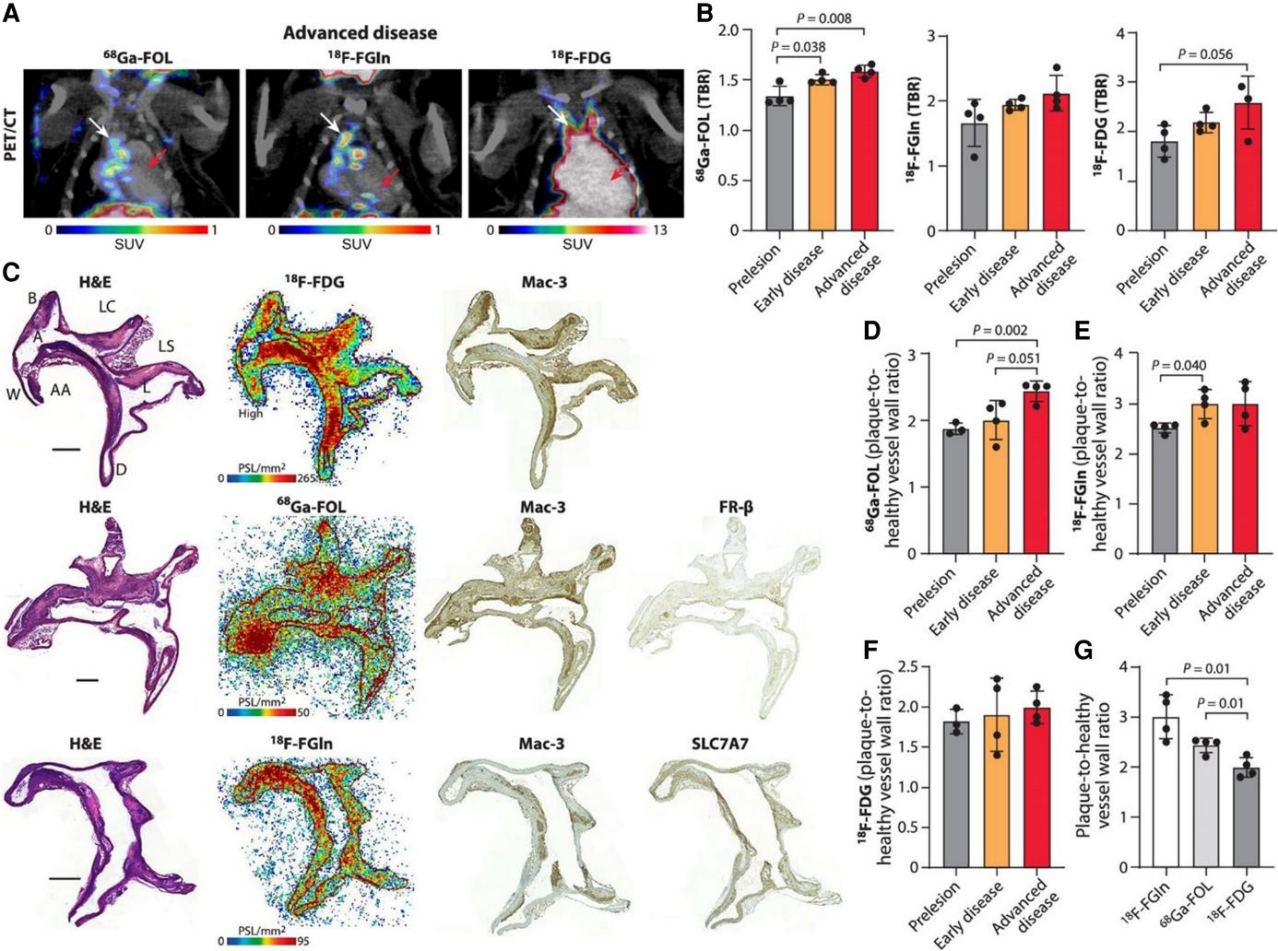
PET studies in mice. (*A*) Representative coronal PET/CT images of mice with advanced atherosclerosis using ^68^Ga-FOL,^18^F-FGln, or ^18^F-FDG. White arrows indicate the aortic arch and red arrows indicate the myocardium. SUV, standardized uptake value. Group size: as in panel *B*. (*B*) Quantification of *in vivo* PET/CT tracer uptake in the aortic arch expressed as target-to-background ratio (TBR = SUV_max, aortic arch_/SUV_mean, blood_, at 40–60 min post-injection). Values are mean ± SD (*n* = 4 mice), except in advanced disease group with ^18^F-FDG (*n* = 3 mice). (*C*) Representative images of haematoxylin–eosin (H&E) staining, autoradiographs, Mac-3 macrophage staining, FR-β and SLC7A7 glutamine transporter staining of consecutive aorta cryosections from a mouse with advanced disease. Black outline sketch denotes the plaque region. Scale bar = 500 µm. A, arch; AA, ascending aorta; B, brachiocephalic artery; D, descending thoracic aorta; L, lesion; LC, left common carotid artery; LS, left subclavian artery; W, vessel wall. PSL/mm^2^, photostimulated luminescence per square millimetre. Group size: as in *D*–*F*. (*D*–*F*) Quantification of autoradiography data expressed as plaque-to-healthy vessel wall ratio. Values are mean ± SD (*n* = 4 mice), except in prelesion group with ^68^Ga-FOL and ^18^F-FDG (*n* = 3 mice). (*G*) Quantification of autoradiography data from advanced disease group expressed as plaque-to-healthy vessel wall ratio showing difference between the tracers. Values are mean ± SD (*n* = 4 mice). *P*-values were calculated using a two-tailed unpaired Student’s *t* test.

We investigated the uptake of ^18^F-FGln and ^68^Ga-FOL in comparison to the widely used tracer, ^18^F-FDG, across the three disease stages in LDLR^−/−^ApoB^100/100^ mice (mouse characteristics summarized in Supplementary material online, *Table S13*). PET/CT images of advanced disease showed uptake of ^18^F-FGln and ^68^Ga-FOL in aortic arch, while with ^18^F-FDG aortic arch uptake was poorly resolved due to high physiological myocardial uptake (*Figure 4A*). To specifically quantify aortic arch uptake while accounting for radioactivity residing in blood, we calculated a TBR comparing aortic arch to vena cava as a representation of the background signal of blood. The average TBR of ^68^Ga-FOL in the aortic arch was significantly higher in both early disease (*P* = 0.04) and advanced disease (*P* = 0.008) compared to prelesion stage (*Figure 4B*). Similarly, the TBR of ^18^F-FGln in the aortic arch showed higher tendency in early disease and advanced disease compared to prelesion stage, although statistical significance was not reached. Likewise, the TBR of ^18^F-FDG in the aortic arch showed a trend of higher values in early and advanced disease compared to prelesion stage without a statistically significant difference. *Ex vivo* gamma counting, using whole aorta rinsed clean of blood, confirmed the results of *in vivo* PET data (see Supplementary material online, *Figure S14A*).

The myocardial uptake of ^18^F-FDG and ^68^Ga-FOL increased during the development of the disease while no difference in the myocardial uptake of ^18^F-FGln was observed between the disease groups (see Supplementary material online, *Figure S14B*). However, in the advanced disease, the myocardial uptake of ^18^F-FGln and ^68^Ga-FOL (both *P* < 0.001) were significantly lower compared to ^18^F-FDG, and the uptake of ^68^Ga-FOL was significantly lower (*P* < 0.01) than that of ^18^F-FGln (see Supplementary material online, *Figure S14B*). The uptake of the tracers in other tissues determined by *ex vivo* gamma counting is listed in Supplementary material online, *Tables S14*–*S16*.

To investigate the precise localization of the tracers uptake in the aorta, a comparative analysis was performed by examining autoradiographs with adjacent histological and immunohistochemical staining. The analysis demonstrated a localization of ^68^Ga-FOL, ^18^F-FGln, and ^18^F-FDG uptake in macrophage-rich lesions in line with the expression profiles of FR-β and SLC7A7 (*Figure 4C*). Especially plaque ^18^F-FGln uptake was clearly higher compared to vessel wall and adventitia in all disease groups (see Supplementary material online, *Figure S14C* and *Table S17*). The plaque-to-healthy vessel wall ratio of ^68^Ga-FOL was significantly higher in advanced disease compared to early disease (*P* = 0.05) and prelesion stage (*P* = 0.002, *Figure 4D*). The plaque-to-healthy vessel wall ratio of ^18^F-FGln was significantly higher in early disease (*P* = 0.04) but not in advanced disease compared to prelesion stage (*Figure 4E*). The plaque-to-healthy vessel wall ratio of ^18^F-FDG was similar in all disease stages (*Figure 4F*). In advanced disease, the plaque-to-healthy vessel wall ratio of ^18^F-FGln and ^68^Ga-FOL were significantly higher compared to ^18^F-FDG (*Figure 4G*). Collectively, these findings indicate that both ^18^F-FGln and ^68^Ga-FOL are promising non-invasive markers that reflect the atherosclerotic burden within the vessels.

### 3.5 *Slc7a7* contributes to foam cell phenotype

**Figure 5:**
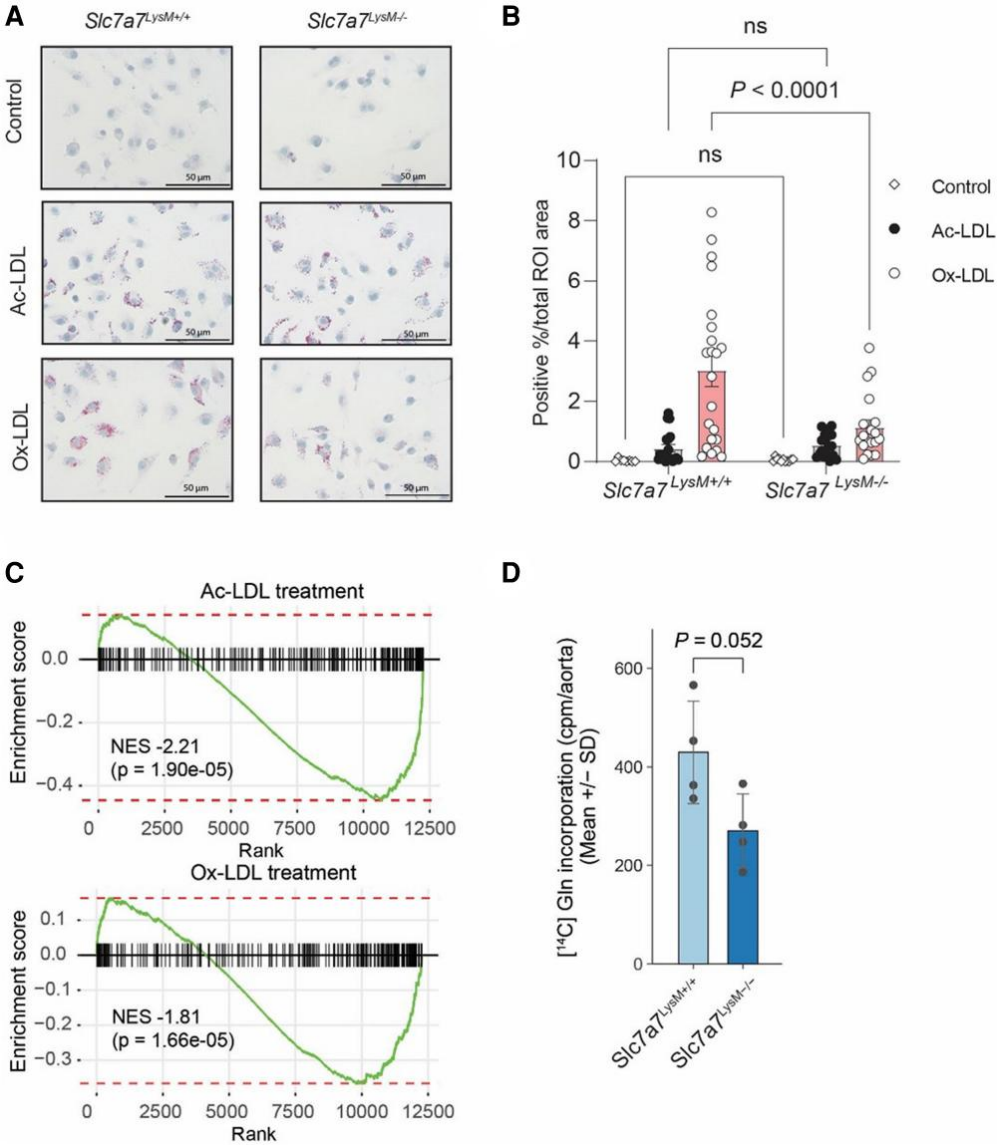
Evaluation of lipoprotein uptake and gene expression in *Slc7a7* knockout macrophages. (*A*) Histological analysis of macrophage uptake of lipoproteins in Slc7a7 knockout and wild type mice. Images depict bone-marrow-derived macrophages (BMDM-s) from control, acetylated LDL (Ac-LDL), and oxidized LDL (Ox-LDL) treated groups for both in *Slc7a7^LysM+/+^* (wild type) and *Slc7a7^LysM−/−^* (knockout) mice. Images are representative of typical results of BMDM-s generated from three mice per genotype. Scale bar: 50 µm. (*B*) Quantification of positive ROI (region of interest) area comparing control, Ac-LDL, and Ox-LDL treatments in *Slc7a7^LysM+/+^* and *Slc7a7^LysM−/−^* macrophages. Each dot represents an individual sample measurement, with the horizontal line indicating the mean and error bars representing standard deviation. The number of measurements for *Slc7a7^LysM+/+^* and *Slc7a7^LysM−/−^*, respectively, was 8 and 11 (Control), 19 and 19 (Ac-LDL), and 22 and 19 (Ox-LDL). Statistical significance is denoted as ‘ns’ (not significant) or by *P*-value (two-way ANOVA followed by Šídák’s multiple comparisons test). (*C*) Gene set enrichment analysis comparing expression of the lipid-associated macrophage gene signature between *Slc7a7*^LysM−/−^ and *Slc7a7*^LysM+/+^ BMDM-s (*n* = 3 per genotype) subjected to either Ac-LDL or Ox-LDL treatment. The plot shows the running enrichment score (ES) and the associated *P*-values for the gene set as it is evaluated across the ranked list of genes. Vertical bars mark the positions of the gene set members within the ranked list. The dotted line indicates the point where the enrichment score reaches its maximum (for positive enrichment) or minimum (for negative enrichment), representing the position of strongest enrichment within the ranked gene list. (*D*) [^14^C]glutamine incorporation in aortas from Ldlr^−/−^ mice transplanted with BM from either *Slc7a7^LysM+/+^* or *Slc7a7^LysM−/−^* mice and fed an atherogenic diet for 11 weeks. Data from individual mice are presented (*n* = 4 mice per group) and summarized as the mean ± SD (cpm, counts per minute). Two-tailed Student’s *t*-test was used for statistical analysis.

The *Slc7a7* gene encodes for a protein that is part of an amino acid transporter system. Specifically, it is a component of the y + L amino acid transport system, which is involved in the transport of cationic amino acids (efflux from the cell) in exchange of large neutral amino acids with sodium (influx into the cell) across the plasma membrane, Gln being one of the preferred neutral amino acid substrates.^56,57^ Among the reported glutamine transporters, scRNA-Seq of the mouse aorta revealed that *Slc7a7* exhibited a tendency for co-expression with *Trem2* (see Supplementary material online, *Figure S11A*), whereas *Slc7a8* was more broadly expressed in macrophages (see Supplementary material online, *Figure S15*). In contrast, the glutamine transporters *Slc1a5*, *Slc38a1*, *Slc38a2*, and *Slc3a2* were ubiquitously expressed across all cell types within the mouse plaque (see Supplementary material online, *Figure S15*). In human coronary artery scRNA-Seq, *SLC7A7* was also specific to macrophages as a cell type, but its expression covered both *TREM2*-positive and *TREM2*-negative subpopulations (see Supplementary material online, *Figure S11B*). Dysfunctional mutations in *Slc7a7* gene lead to lysinuric protein intolerance, a rare metabolic disorder that results from defective transport of cationic amino acids.^58^ The *Slc7a7* knockout mouse model recapitulates this phenotype.^59^ However, despite these findings, the understanding of *Slc7a7*'s role in macrophages is still limited. Notably, *Slc7a7* has been identified as crucial for macrophage viability and efferocytosis in zebrafish.^60^ While it promotes an anti-inflammatory state in cultured macrophages,^61^ its role in lipid loading associated with LAMs remains uncharacterized. To investigate this, we differentiated BM-derived macrophages from myeloid-specific (LysM-Cre) *Slc7a7* knockout mice (*Slc7a7^LysM−/−^*), which exhibited a 73–88% reduction in *Slc7a7* expression compared to control mice (*Slc7a7*^LysM+/+^; Supplementary material online, *Figure S16*). We then exposed control and *Slc7a7^LysM−/−^* macrophages to acetylated LDL (Ac-LDL) and oxidized LDL (Ox-LDL) to assess foam cell formation and to identify global changes in gene expression. In wild type cells, both forms of LDL upregulated *Slc7a7* mRNA expression (see Supplementary material online, *Figure S16*). Oil Red O staining results indicated impaired Ox-LDL uptake in *Slc7a7^LysM−/−^* BM-derived macrophages compared to wild type (*Figure 5A* and *B*). No difference was evident between the genotypes for Ac-LDL uptake, potentially due to the considerably lower overall uptake signal of Ac-LDL in this experiment (*Figure 5B*). Based on RNA-Seq, deletion of *Slc7a7* attenuated the transcriptional signature associated with LAMs in both Ac-LDL and Ox-LDL treatments (*Figure 5C*). Finally, in a BM transplantation experiment, LDLR-deficient mice that received *Slc7a7^LysM−/−^* BM showed a trend (*P* = 0.052) towards reduced glutamine uptake in aorta after 11 weeks of HFD, relative to recipients of *Slc7a7^LysM+/+^* BM (*Figure 5D*). Plasma cholesterol and triglyceride levels showed no significant differences between the groups (cholesterol: *Slc7a7^LysM+/+^* 485.8 mg/dL, *Slc7a7^LysM−/−^* 532.3 mg/dL, *P* = 0.26; triglycerides: *Slc7a7^LysM+/+^* 76.4 mg/dL, *Slc7a7^LysM−/−^* 77.2 mg/dL, *P* = 0.91), indicating that the observed results were not influenced by lipid levels. Collectively, these findings indicate that the loss of *Slc7a7* impedes lipid uptake in macrophages and suggests a significance of glutamine metabolism in foam cell formation.

## 4. Discussion

Macrophages play a major role in the pathogenesis of atherosclerosis. The recent development and rapid progress of single-cell technologies have enabled comprehensive mapping of the wide range of cell types and their phenotypes present in atherosclerotic plaques, including comparison between mouse and human.^4,5^ These studies have identified three main macrophage populations within atherosclerotic plaques, including proinflammatory, LAM, and resident macrophages.^1^ A better understanding of the cell phenotype and function of atherosclerosis-associated populations, along with their correlation with disease progression, clinical features, and future events, may help guide future strategies to predict and mitigate atherosclerosis complications. To this end, we provide a comprehensive identification of disease associated macrophages in three major tissues implicated in the pathogenesis of atherosclerosis. We identified a signature of 388 genes that are shared across both mouse and human plaques. Among them, 164 represent membrane-bound receptor or metabolic proteins, making them amenable for non-invasive disease detection and monitoring.

Trem2+ LAMs have been shown to be widely distributed across healthy and diseased human tissues.^62^ TREM2, a membrane protein expressed on immune cells, has been implicated in both neurodegenerative diseases and cancer, with studies suggesting therapeutic potential in targeting its activity.^63,64^ In line with this, the cerebrospinal fluid levels of sTREM2 have been associated with Alzheimer’s disease,^65^ whereas recently also serum levels of sTREM2 have been associated with MASH^52^ and cardiovascular death.^54^ In this study, we provide evidence that sTREM2 levels are also correlated with plaque vulnerability, notably exhibiting a heightened capacity to distinguish symptomatic patients from asymptomatic. Importantly, tissue TREM2 levels in early and advanced lesions, and particularly in the liver, showed significant correlations with plasma levels, suggesting that circulating sTREM2 may serve as a proxy for underlying tissue-specific changes. Supporting the liver’s role in this axis, plasma sTREM2 levels also correlated with cholesterol and triglyceride levels, a finding supported by a recent study analysing plasma proteins in a cohort of 35 000 Icelanders.^66^ This pattern suggests a complex interplay between sTREM2 and lipid metabolism, as well as inflammatory processes in the body and motivates future studies to explore the relationship between sTREM2 and tissue lipid content Mouse studies investigating the role of TREM2 in atherosclerosis (recently reviewed in ref.^67^) have shown that haematopoietic or global TREM2 deficiency enhances necrotic core formation in early atherosclerosis, whereas TREM2 agonism reduces it.^55^ However, this effect appears to be model-dependent, as another study reported that macrophage-specific TREM2 deficiency led to reduced lesion formation.^68^ Additionally, TREM2 agonist antibody was shown to improve plaque stability by promoting macrophage survival, smaller necrotic core, and greater collagen deposition.^69^ These findings also suggest that modulating TREM2 activity could be a promising strategy for improving cardiovascular outcomes in patients with elevated lipid levels and inflammatory markers.

Traditional imaging methods like computed tomography (CT) and magnetic resonance (MR) mainly offer anatomical insights with limited biochemical and functional data. PET imaging, however, offers precise biochemical quantification, high sensitivity, and clinical adaptability, making it a preferred method for tracking inflammation in CVDs.^70,71^ The predominantly used PET tracer is ^18^F-FDG, which, despite its widespread use and availability, has drawbacks. As a glucose analogue, it is accumulating in metabolically active tissues such as myocardium, causing background noise.^72^  ^18^F-FDG's effectiveness can also be influenced by fasting, blood glucose, insulin levels, and certain drugs, making it less suitable for diabetic patients with hyperglycaemia.^73^ To address this limitation, we have previously described the radiosynthesis of ^18^F-FGln and ^68^Ga-FOL and demonstrated lower myocardial uptake compared ^18^F-FDG.^27,28^ Here, this analysis was extended to provide a more detailed time course analysis of the accumulation of resident (^68^Ga-FOL) and LAM (^18^F-FGln) macrophages. Our analysis demonstrated that both tracers reflect the disease associated macrophage burden with minor differences in the dynamics. Specifically, ^18^F-FGln demonstrated slightly higher accumulation and plaque-to-healthy wall ratio during the early disease stages compared to ^68^Ga-FOL, indicating temporal variations between the two. Although the overall target-to-background ratio for ^18^F-FGln was higher, ^68^Ga-FOL showed more promise in detecting early disease and differentiating between mid and advanced stages, as the difference between the disease and pre-disease states with 18F-FGln was not significant. We acknowledge that the small sample sizes in this study limit the statistical power regarding temporal differences between tracers, and larger studies are needed to validate these findings.

Glutamine has been shown to affect macrophage polarization, where high glutamine uptake and glutaminolysis are associated with an anti-inflammatory and efferocytic phenotype.^35,74,75^ Notably, around one-third of the carbon in tricarboxylic acid metabolites and more than half of the nitrogen in uridine diphosphate *N*-acetylglucosamine (UDP-GlcNAc) synthesis in M2-like macrophages originates from glutamine, emphasizing its critical role in macrophage polarization. In line with this, transient glutamine deprivation has been observed to adversely impact the M2 activation programme. Previous *in vitro* studies have proposed that enhanced glutamine accumulation could be due to increased expression of a glutamine transporter, *Slc1a5*.^75^ Macrophages express multiple glutamine transporters, including *Slc1a5*, *Slc3a2*, *Slc38a1*, and *Slc38a2*—also found in other plaque cell types—and *Slc7a7*, which is restricted to macrophages. Our studies also support that *Slc7a7* contributes to macrophage foam cell formation and expression of the LAM gene signature. This aligns with the requirement of Slc7a7 for efferocytosis and the maintenance of an anti-inflammatory state in macrophages,^60,61^ as well as findings from patients with *SLC7A7* mutations, which results in altered inflammatory responses and impaired phagocytosis by macrophages.^76,77^ While our findings highlight the importance of *Slc7a7*, further research should include other ubiquitously expressed glutamine transporters to fully understand the role of glutamine metabolism in atherosclerosis.

In conclusion, our findings present a novel set of potential biomarkers for atherosclerosis. Considering the pivotal role of macrophages in atherosclerosis, PET imaging offers a non-invasive means to quantify specific disease markers. When integrated with genetic screening results and liquid biopsy biomarker data, this approach holds potential for formulating personalized therapeutic interventions. Additionally, it could be instrumental in refining patient selection for immunotherapy and facilitating its subsequent monitoring. We anticipate that the macrophage markers we have identified will deepen our understanding of the pathophysiology underlying atherosclerosis and guide future research endeavours.

Translational perspective In this study, we characterized macrophage-specific biomarkers and their roles in atherosclerosis using advanced imaging and genomic techniques. The identification of soluble TREM2 as a biomarker for differentiating symptomatic from asymptomatic atherosclerosis could significantly enhance early diagnosis and patient stratification in clinical settings. Additionally, exploring Folr2 and Slc7a7 as positron emission tomography tracers provides a promising avenue for non-invasive assessment of disease burden, potentially allowing for better monitoring of disease progression and treatment efficacy. These findings suggest practical applications in improving personalized treatment plans and could lead to earlier therapeutic interventions, thereby potentially reducing the incidence of severe cardiovascular events.

## Supplementary Material

cvaf210_Supplementary_Data

## Data Availability

The raw sequencing data have been deposited in NCBI GEO under accessions GSE254395 (reviewer token alsraieejvizhax), GSE254396 (reviewer token ipqxskykrdclpsx), and GSE205929 (TRAP-Seq), and GSE254398 (BMDM RNA-Seq; reviewer token qradsumodnsvrqf). BiKE transcriptomic dataset is available from NCBI GEO with accession number GSE21545.
