# Supplementary material for: Single-cell to pre-clinical evaluation of Trem2, Folr2, and Slc7a7 as macrophage-associated biomarkers for atherosclerosis
Source: Cardiovasc Res. 2025 Nov 7;121(16):2503–19. doi: 10.1093/cvr/cvaf210 (PMC12713647; doi:10.1093/cvr/cvaf210)
Supplement: cvaf210_Supplementary_Data [file cvaf210_supplementary_data.zip › Supplementary_Figures R1.docx]

**Supplementary Figures**


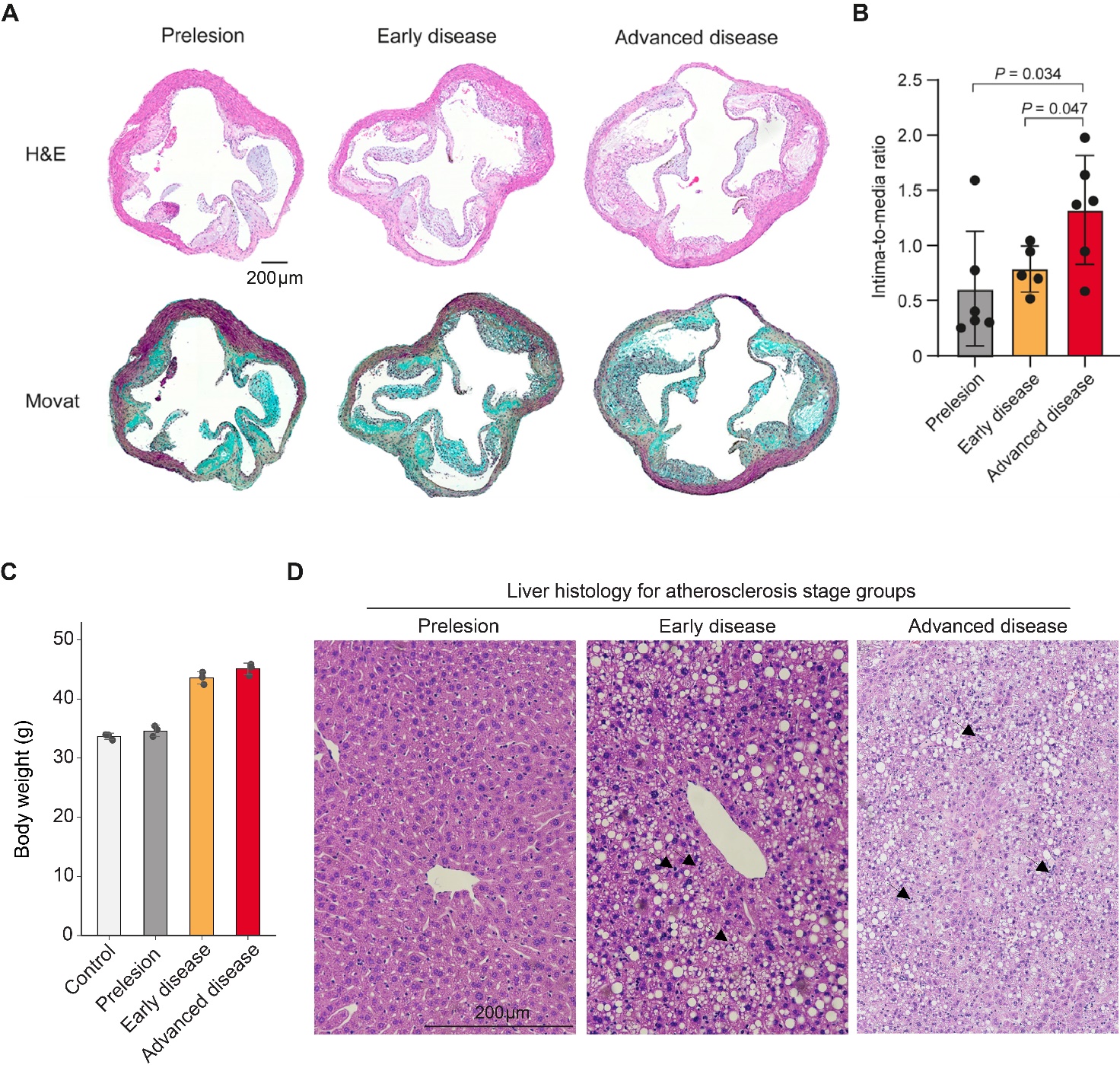


**Supplementary Figure 1.** Pathological effects of the atherosclerosis disease stage course. **(A)** Aortic root sections from different disease stages stained with Hematoxylin-Eosin (H&E) and modified Movat’s pentachrome (Movat). Images are representative of typical results from the samples summarized in panel B. **(B)** Aortic root plaque size at different disease stages as determined by the intima-to-media ratio. *P* values were calculated using the two-tailed unpaired Student’s *t* test. Sample size: 6 mice per group (except early disease, n = 5). **(C)** Mouse body weight by disease stage group for the mice used in scRNA-Seq (n = 3 mice per group). **(D)** Liver sections stained with H&E. Representative sections from a minimum of 3 mice observed per group (atherosclerosis disease stage). Arrowheads indicate infiltration and activation of immune cells. In panels B and C, values are mean ± SD. Scale bars = 200 µm.


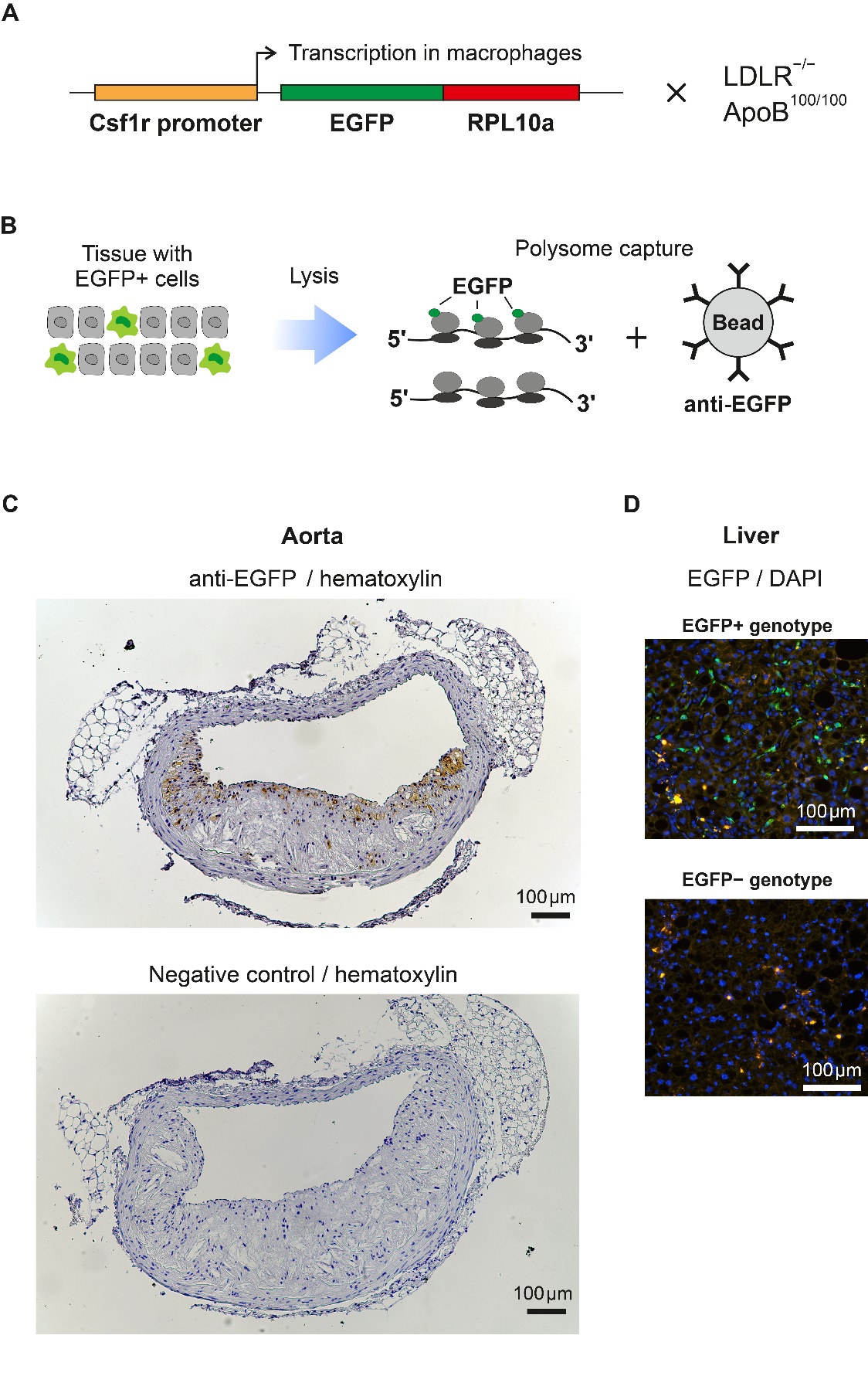


**Supplementary Figure 2. (A)** The TRAP-Seq construct introduced into mice for macrophage-specific expression EGFP-tagged ribosomal protein RPL10a. For atherosclerosis studies, TRAP-Seq mice were crossed with LDLR^−/−^ ApoB^100/100^ hypercholesterolemic mice. **(B)** Overview of immunoprecipitation used in TRAP-Seq to enrich for cell type-specific mRNA from bulk tissue lysate. **(C)** Immunohistochemical staining of mouse aorta with anti-EGFP antibody (top panel) or control IgG (bottom panel). Protein signal (brown) is shown with hematoxylin counterstaining (blue). **(D)** Immunofluorescence imaging of EGFP in livers of EGFP+ TRAP-Seq mice and EGFP− mice. Images show EGFP (green) overlaid with DAPI (blue). For panels C-D, images are representative of typical results of sections from n = 2 mice per genotype. Scale bars = 100 µm.


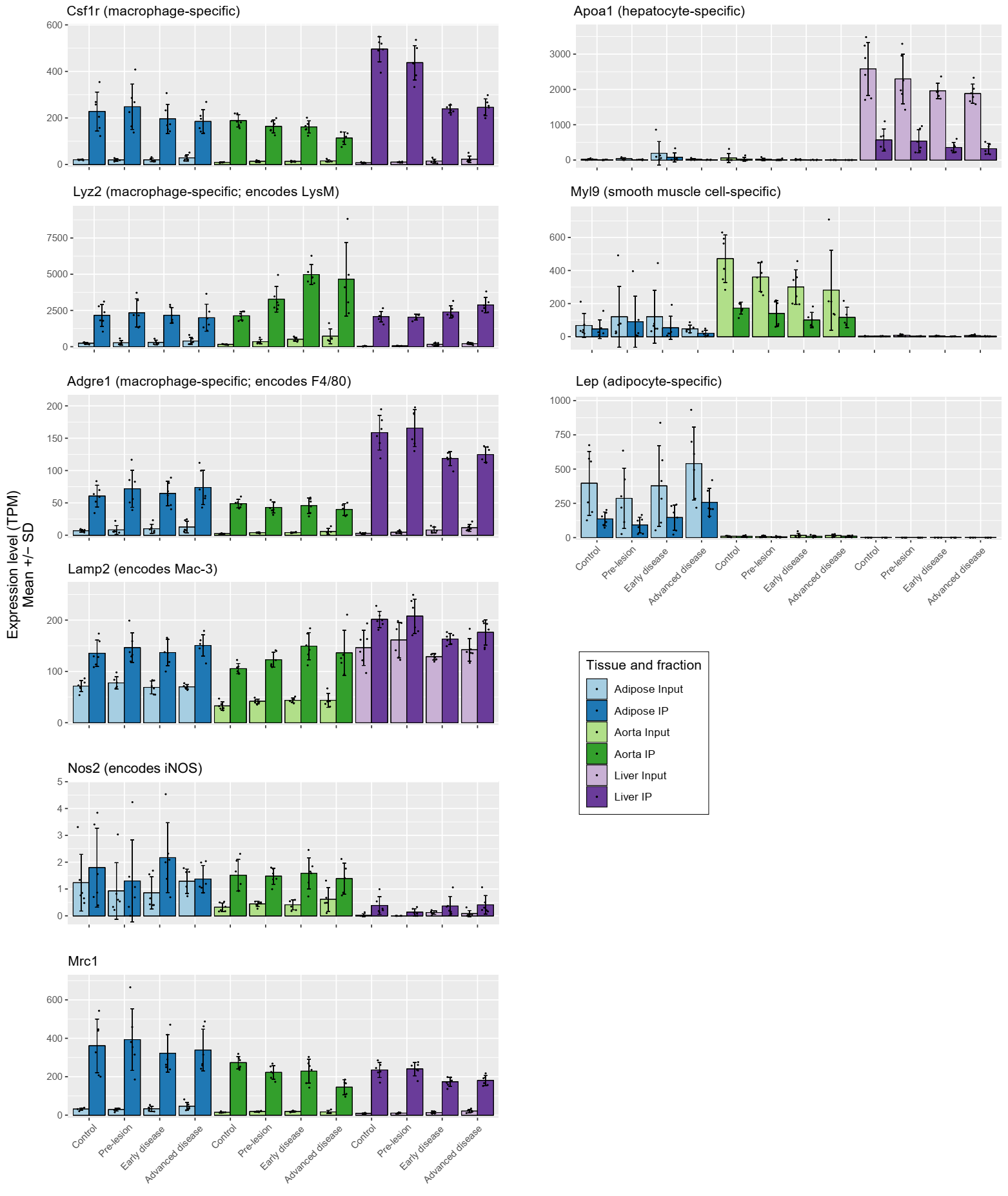


**Supplementary Figure 3.** Expression of macrophage and tissue specific markers in the TRAP-Seq input and EGFP-immunoprecipitated (IP) samples. TPM: transcripts per million. Sample size: 6 mice per group.


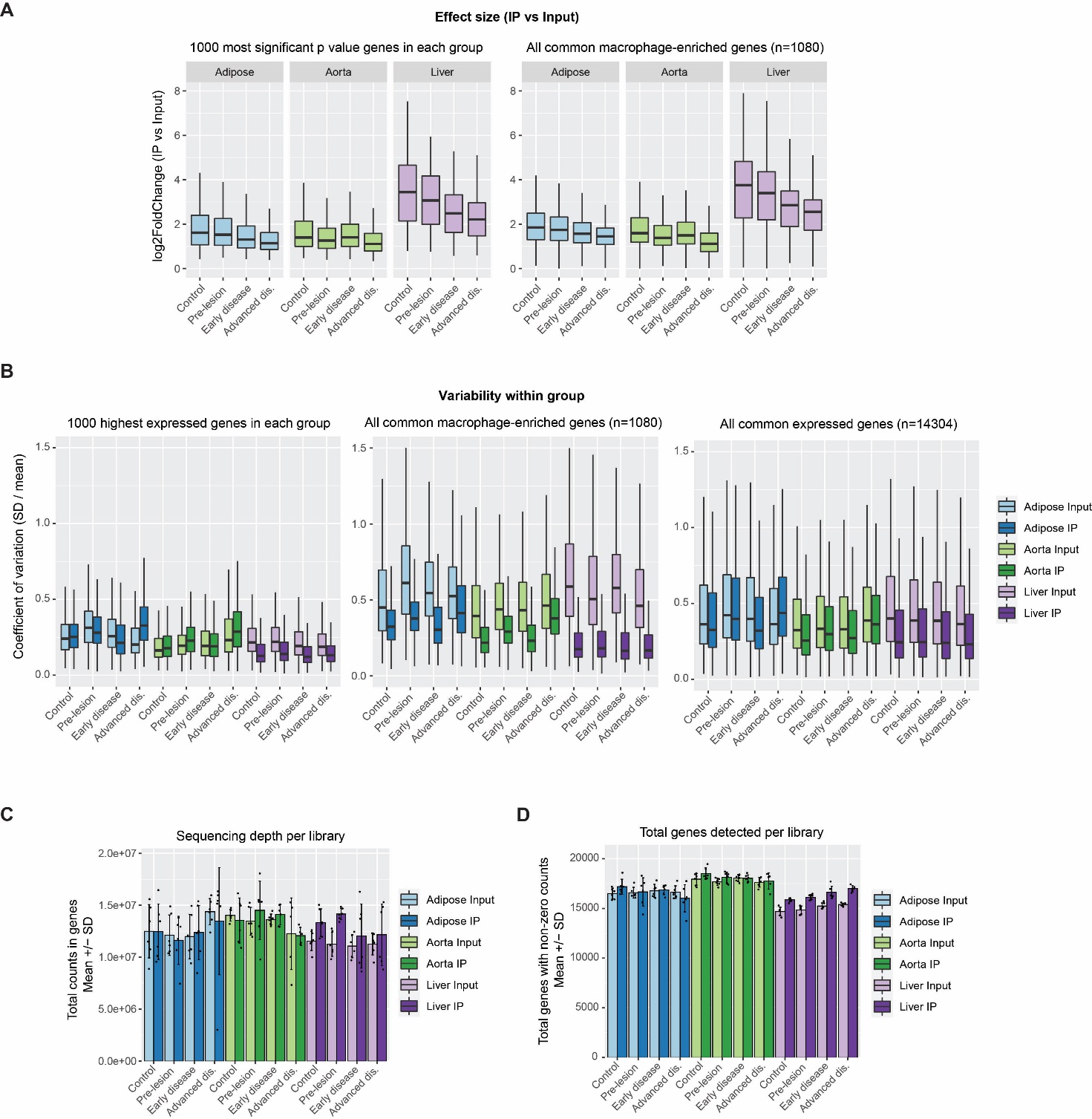


**Supplementary Figure 4.** Comparison of TRAP-Seq assay performance between adipose, aorta and liver tissue. **(A)** TRAP-Seq enrichment effect size (IP vs Input ratio) distribution for top 1000 most statistically significantly enriched genes (selected separately in each experimental group; left panel), or all between-tissue shared macrophage-enriched genes (right panel; n = 1080 genes from Figure 1C). **(B)** Coefficient of variation distribution for the top 1000 highest expressed genes (selected for each group separately; left panel), for all between-tissue shared macrophage-enriched genes (middle panel; n = 1080 genes from Figure 1C), and for all genes considered expressed for differential analysis purposes (right panel; n = 14304 genes). The coefficient of variation was calculated as S.D. / mean from transcripts per million-normalized gene expression. **(C)** Total number of sequencing counts assigned to genes, a measure of library sequencing depth. **(D)** Total number of genes detected with > 0 counts. The transcriptome annotation consisted of 31053 genes.


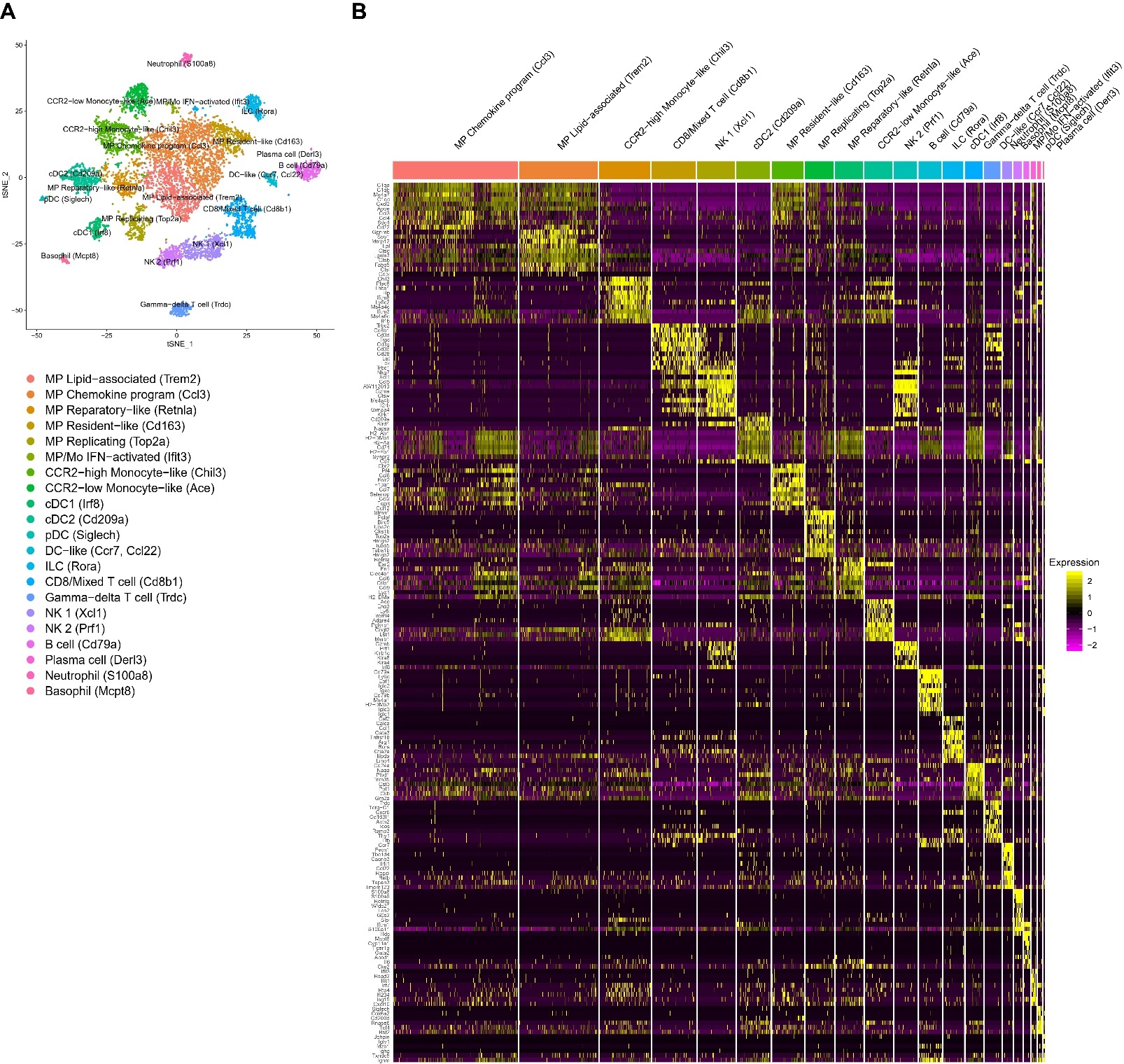


**Supplementary Figure 5. (A)** tSNE projection of the scRNA-Seq profiles of Cd45+ immune cells from the integrated analysis of aorta and adipose tissue (3 mice per disease stage group), grouped into 21 manually annotated clusters. **(B)** Top differentially expressed genes among Cd45+ immune cell clusters from panel A. Row-normalized single-cell gene expression is shown.


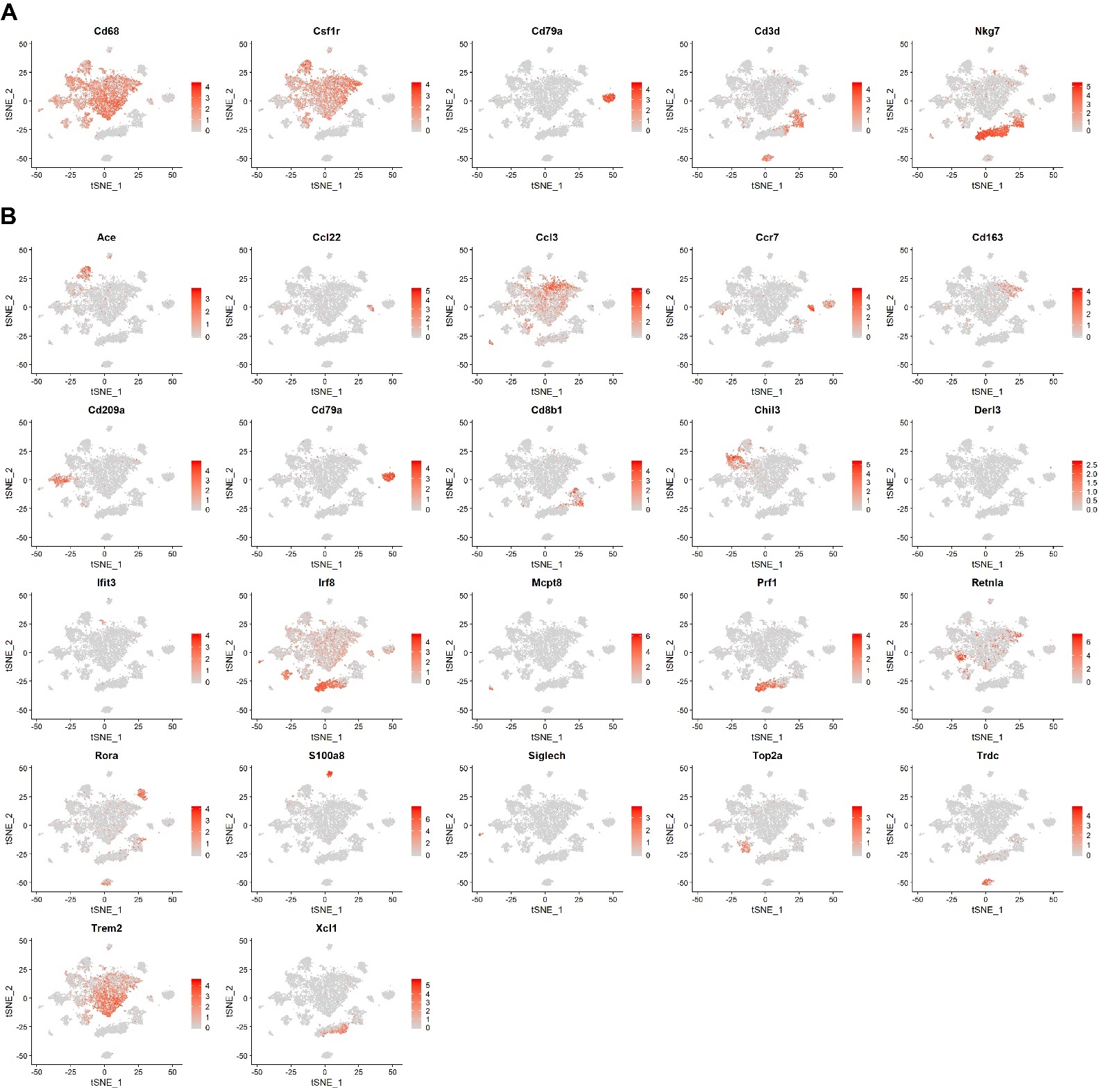


**Supplementary Figure 6.** Gene expression tSNE plots from the integrated analysis of aorta and adipose tissue Cd45+ immune cells (3 mice per disease stage group). **(A)** Genes used to annotate general cell type. **(B)** Marker genes used for cell subtype naming (subtypes shown in Supplementary Figure 5A).


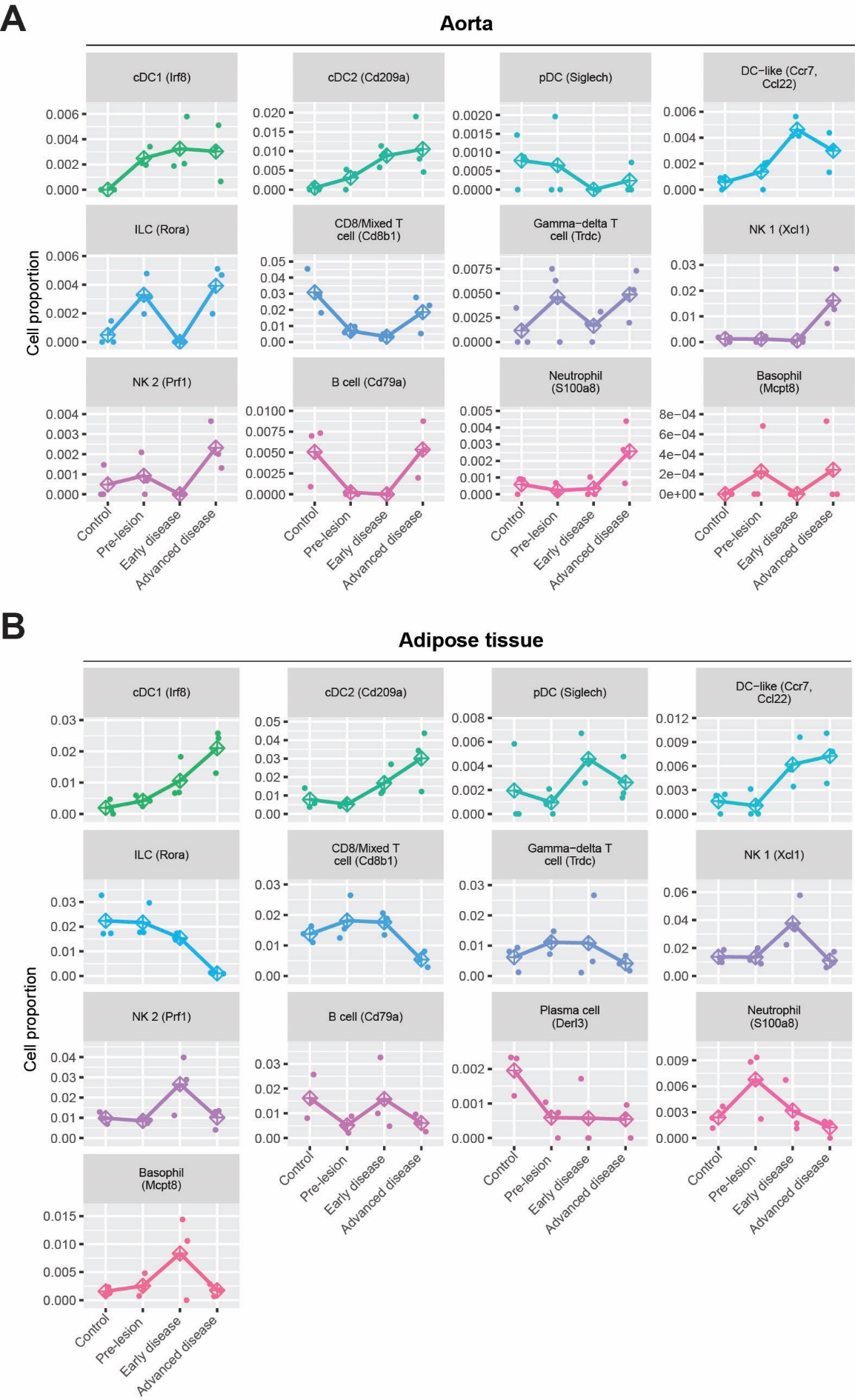


**Supplementary Figure 7.** Cell population proportion within Cd45+ leukocytes in aorta **(A)** and adipose tissue **(B)** during the atherosclerosis disease stage course. The data are presented separately for each of the three biological replicates. Proportions for monocyte/macrophage subpopulations are shown separately (Figure 2C).


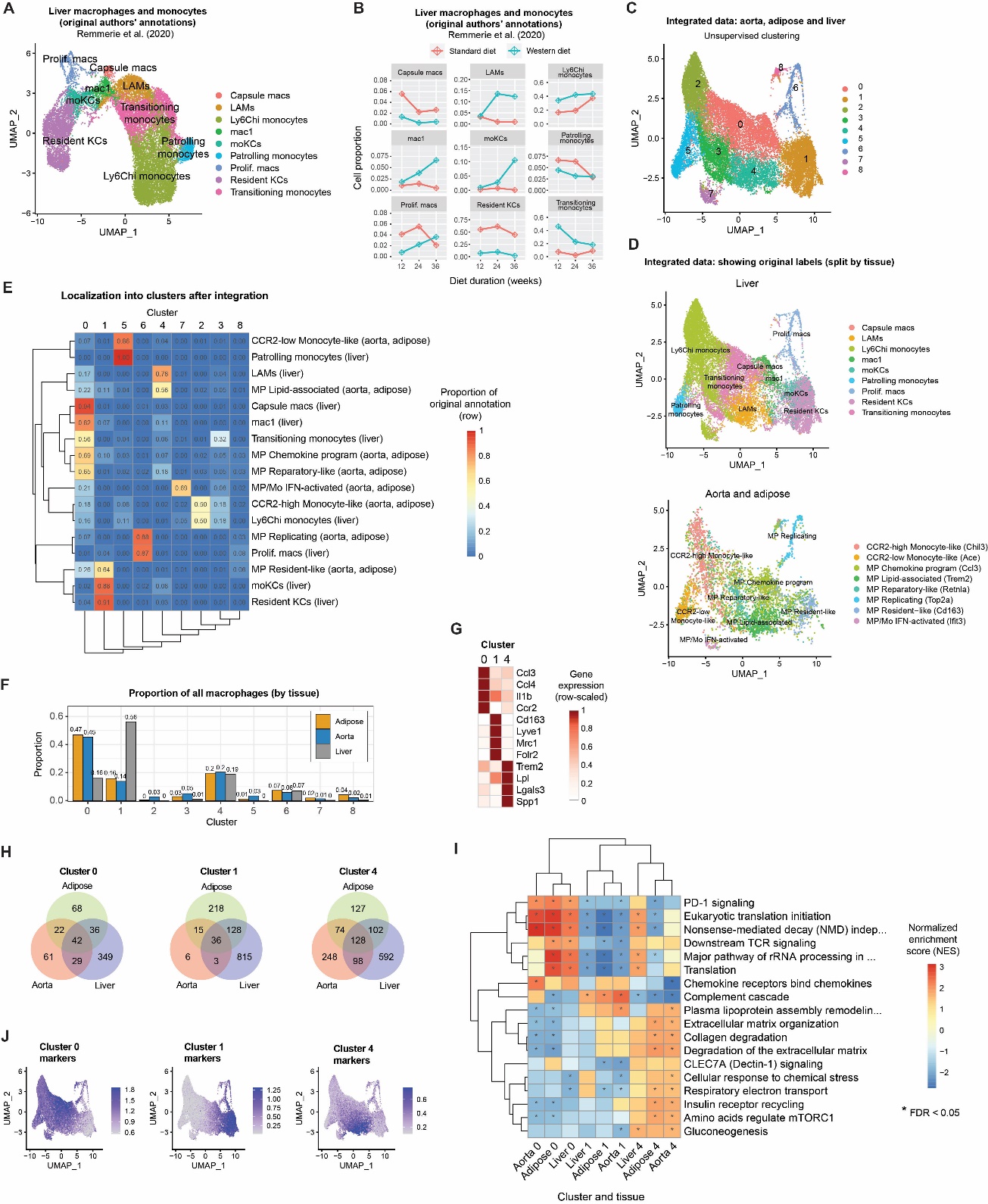


**Supplementary Figure 8.** Integration of aorta and adipose tissue scRNA-Seq macrophages/monocytes with previously published liver macrophages/monocytes (Remmerie *et al.*, 2020; PMID 32888418). **(A)** Overview of the liver cells from Remmerie *et al.*, (2020) showing the original authors’ subtype annotations. **(B)** Liver cell subtype proportions as a function of diet type and duration. Proportions are calculated out of all macrophage/monocyte cells per sample using the original authors’ annotations. **(C)** Integration of aorta, adipose and liver cells, followed by unsupervised clustering. **(D)** Original annotations for liver and adipose/aorta cells visualized in the integrated UMAP plot. **(E)** Localization of original annotations into clusters of the integrated analysis. For each original annotation (row), the proportion of cells falling into each integrated cluster (0-8) is shown. **(F)** Distribution of macrophages (monocytes excluded) into integrated clusters. **(G)** Expression of macrophage subtype marker genes for the 3 major clusters containing macrophages (integration clusters 0, 1 and 4). Cluster pseudobulk gene expression is shown scaled per gene from 0 to 1 (maximum observed). **(H)** Per-tissue marker genes of each integrated macrophage cluster. For each tissue-cluster combination, markers were calculated relative to all other macrophages of the same tissue. **(I)** Reactome pathway enrichment for each tissue-cluster combination. Markers from panel H were analyzed using fgsea. **(J)** Gene set expression scores for the 3-way shared cluster markers from panel H. Gene set activity was calculated using the Seurat module score function and is shown in the adipose–aorta–liver integrated UMAP plot (as in panels C-D). For all panels, the aorta and adipose tissue encompasses 4 disease stages with 3 mice each, and the liver data spans 6 diet-timepoint combinations with 1 mouse each.


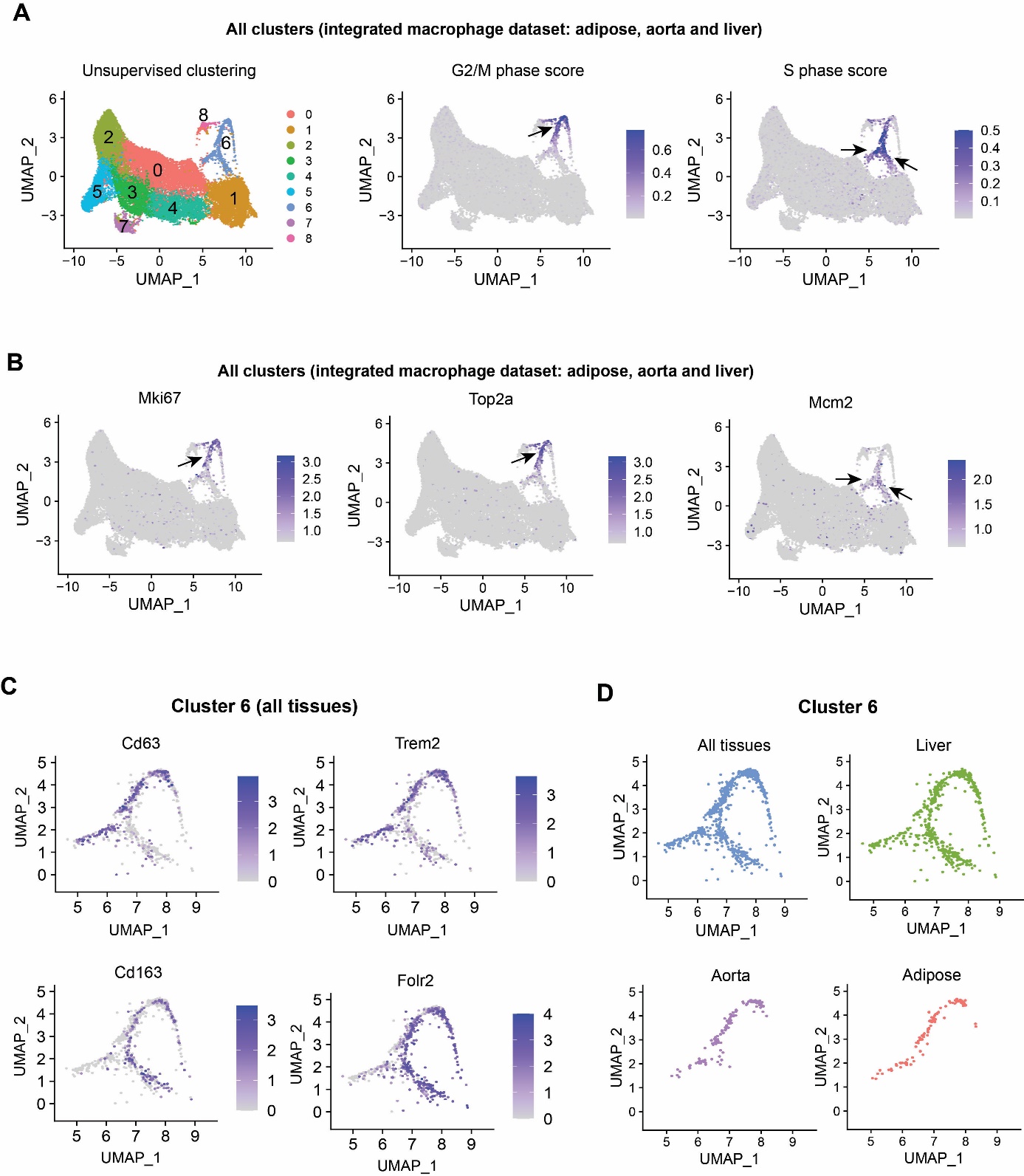


**Supplementary Figure 9.** Additional analysis of proliferating macrophages from the integrated scRNA-Seq analysis of aorta, adipose tissue and liver macrophages. The liver scRNA-Seq data was originally published by Remmerie *et al.,* 2020 (PMID 32888418). **(A)** Cross-tissue integrated UMAP plot showing cell cycle phase scores. Arrowheads highlight regions within cluster 6 with high G2/M phase or S phase scores. **(B)** Gene expression of selected cell cycle marker genes. Arrowheads denote regions of high gene expression. **(C)** Expression of selected macrophage subpopulation marker genes within cluster 6 (LAM markers *Cd63* and *Trem2*, and resident-like macrophage markers *Cd163* and *Folr2*). **(D)** Cluster 6 cells split by tissue-of-origin. For panels C-D, the coordinate space is as in panels A-B, but only cluster 6 cells are plotted. For all panels, the scRNA-Seq of aorta and adipose tissue encompasses 4 disease stages with 3 mice each, and the liver data spans 6 diet-timepoint combinations with 1 mouse each. LAM, lipid-associated macrophage.


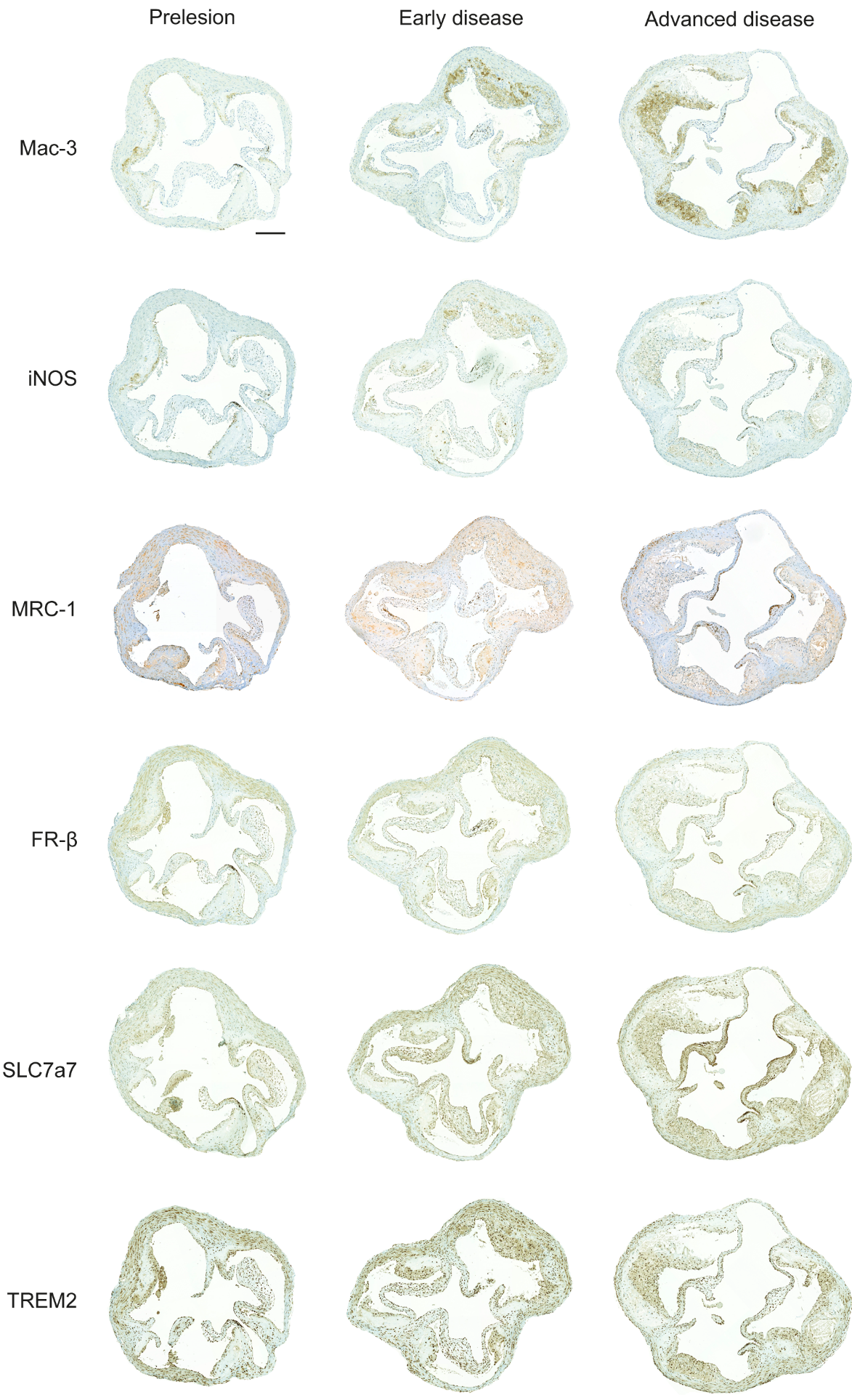


**Supplementary Figure 10.** Representative micrographs of mouse aortic root sections from different stages of atherosclerosis immunohistochemically stained with different macrophage markers. Positive immunostaining is shown in brown and nuclei are shown in blue (hematoxylin). Images are representative of typical results from the samples Supplementary Figure 1B. Scale bar = 200 µm.


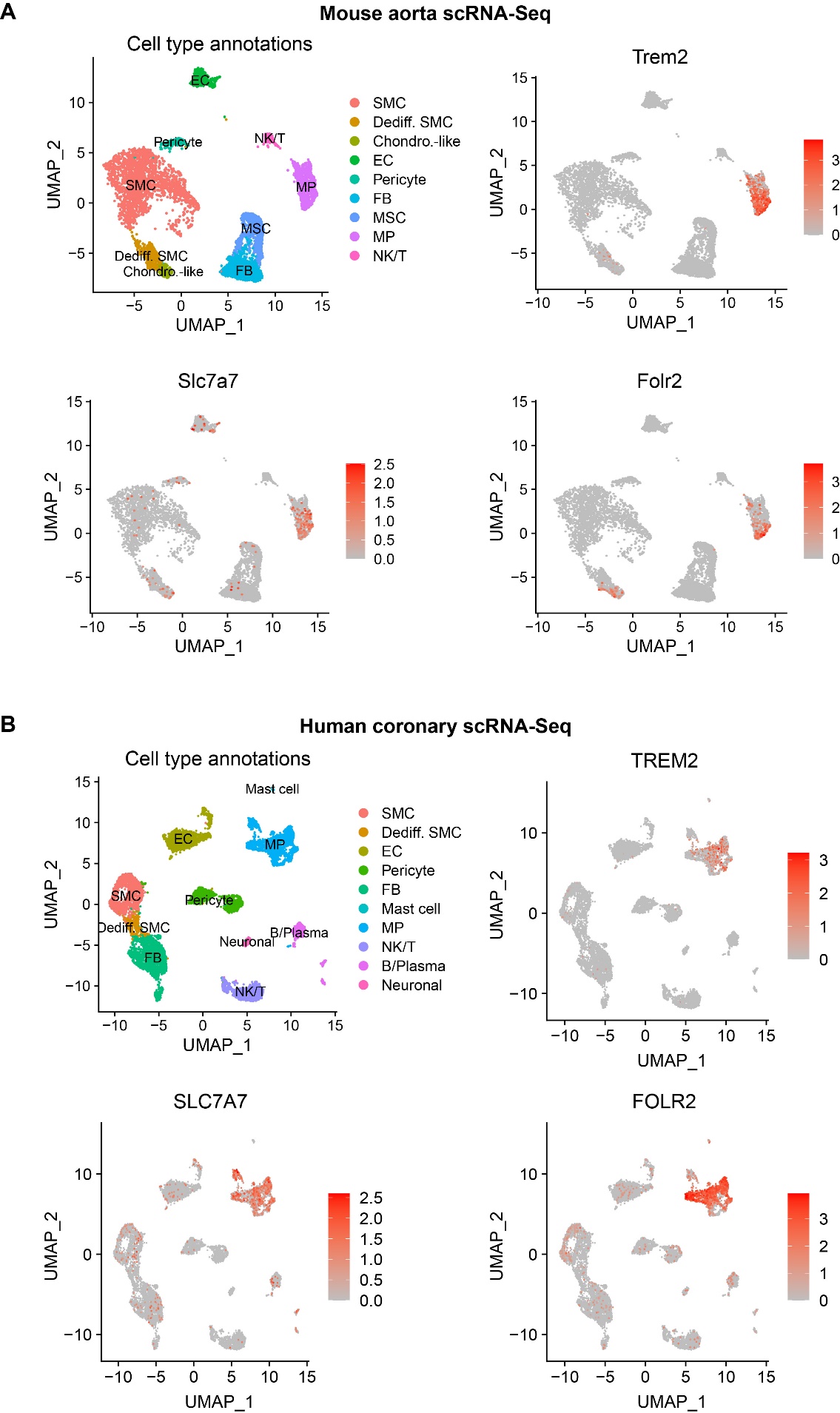


**Supplementary Figure 11.** scRNA-Seq gene expression of *Trem2*, *Slc7a7* and *Folr2* plotted across all major cell types of atherosclerotic mouse aorta **(A)** and human coronary artery **(B)**. The mouse plaque scRNA-Seq profiles encompass 4 disease stages with 3 mice each, as described in Örd *et al*., 2023 (PMID 37060905). The human atherosclerotic coronary artery scRNA-Seq is described in Wirka *et al*., 2019 (PMID 31359001) and includes data from 4 patients.


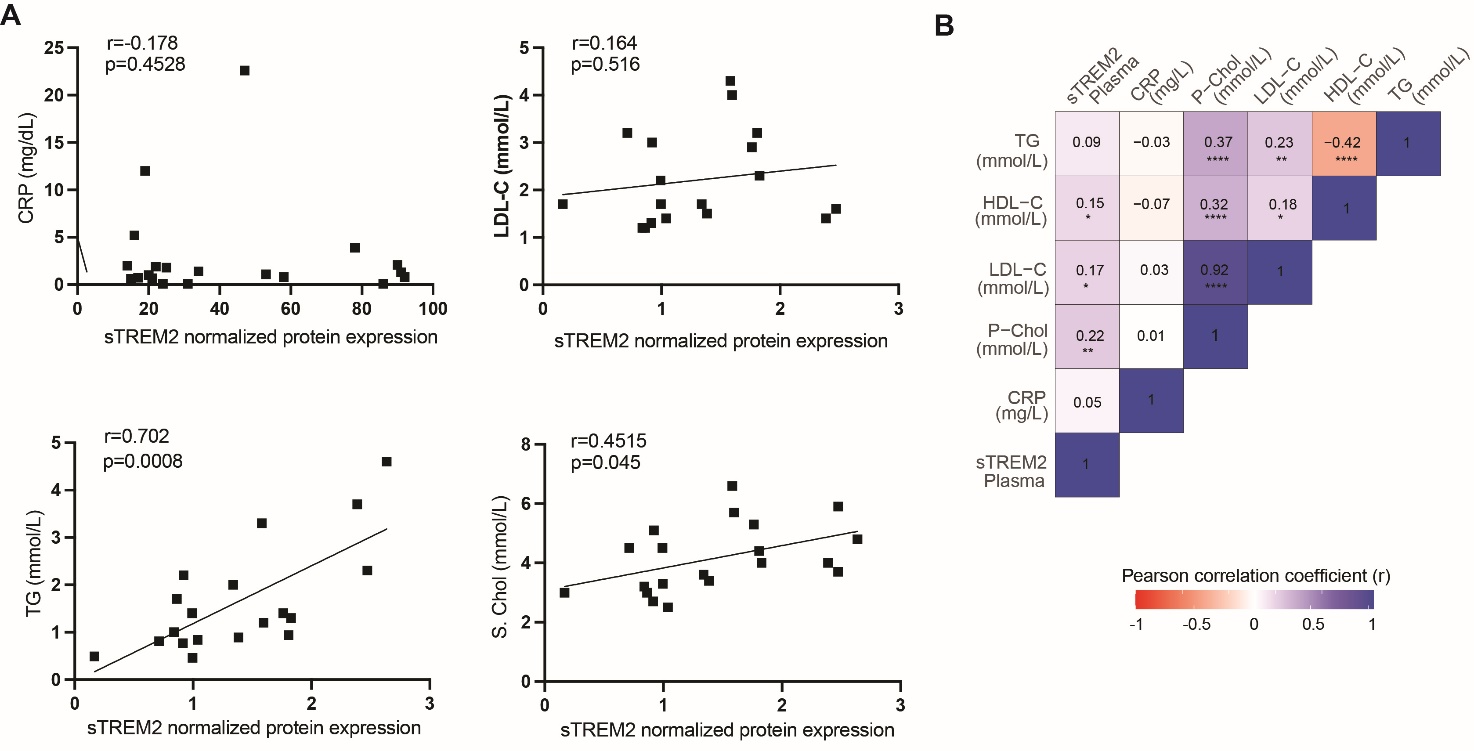


**Supplementary Figure 12**: (**A**) Correlations between sTREM2 normalized protein expression and CRP or lipid levels in 20 individuals of the BiKE cohort. Each plot displays individual data points along with a regression line indicating the relationship between the variables. Pearson correlation coefficients (r) and corresponding p-values are provided for each correlation. (**B**) Pearson correlation matrix showing relationships between plasma sTREM2, C-reactive protein (CRP), serum cholesterol (S-Chol), low-density lipoprotein cholesterol (LDL-C), high-density cholesterol (HDL) and triglycerides (TG) in 165 coronary artery disease patients from the STARNET cohort.


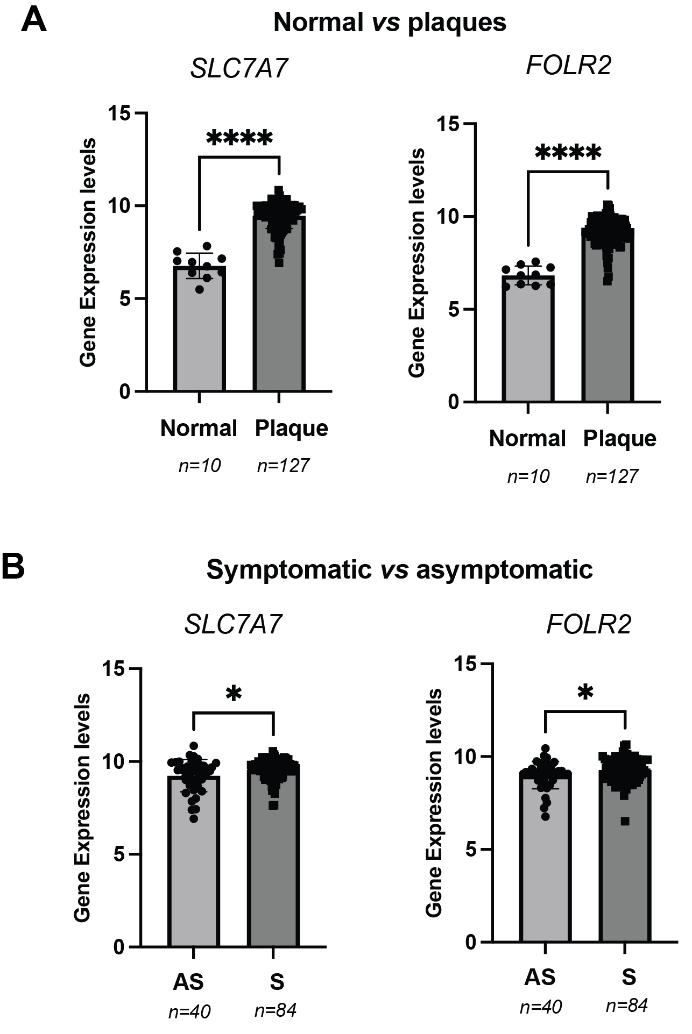


**Supplementary Figure 13.** (**A**) The bar graphs display expression levels of the genes *SLC7A7* and *FOLR2*, comparing normal arterial tissue (n = 10 individuals) to atherosclerotic plaque tissue (n = 127 individuals). The y-axis represents gene expression levels. Statistical significance is denoted by four asterisks (****), indicating a p-value < 0.0001, as determined by a two-sided Student’s t-test with unequal variance. (**B**) Comparison of gene expression in symptomatic versus asymptomatic carotid stenosis patients. The graphs show expression levels of *SLC7A7* and *FOLR2* among patients with asymptomatic (AS, n = 40) and symptomatic (S, n = 84) carotid stenosis. Gene expression levels are plotted on the y-axis. Statistical significance is indicated by a single asterisk (*), representing *P* < 0.05, based on a two-sided Student’s t-test with unequal variance.


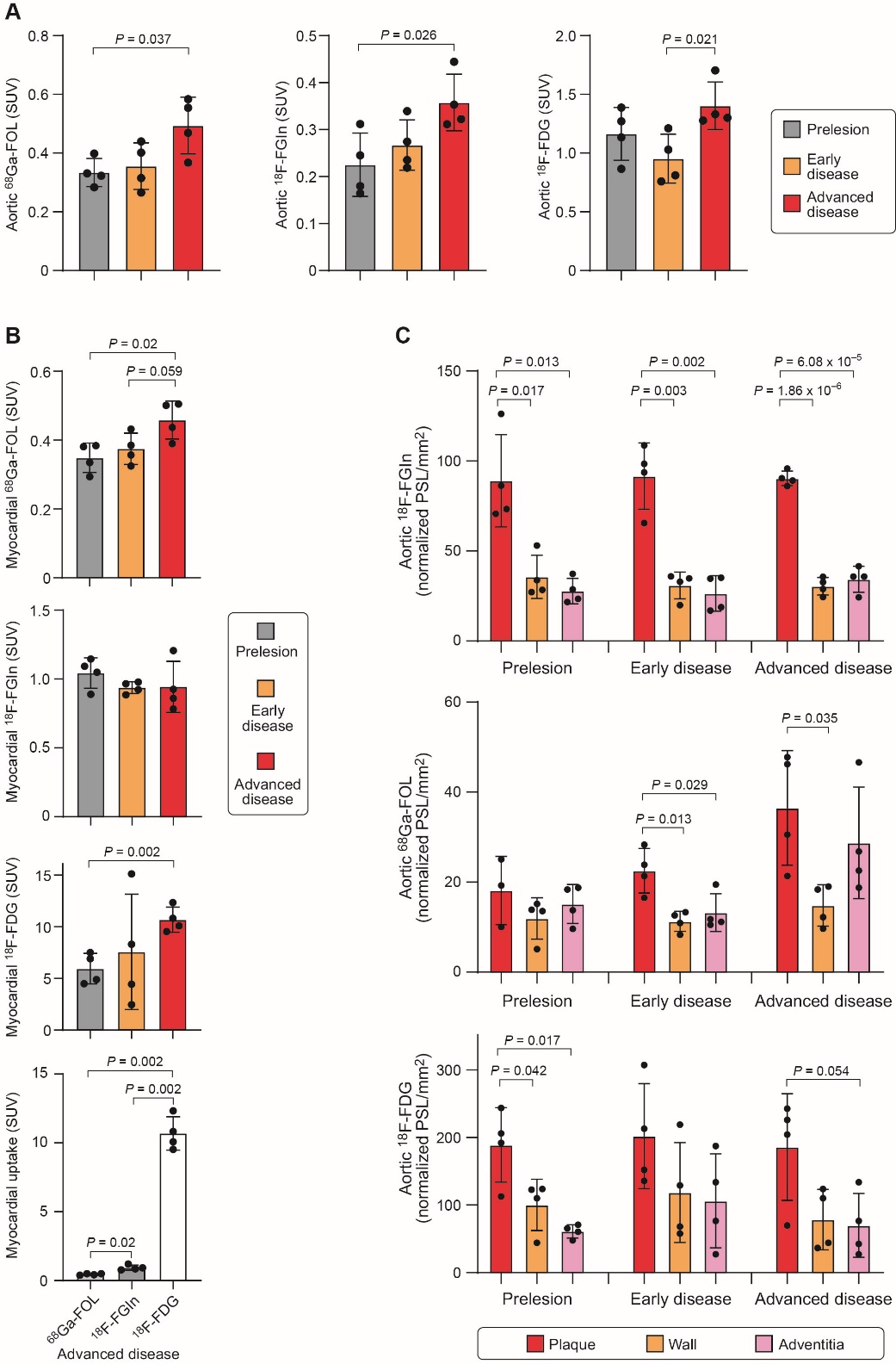


**Supplementary Figure 14.** (**A**) Quantification of ^68^Ga-FOL ,^18^F-FGln and ^18^F-FDG tracer uptake in the whole aorta by *ex vivo* gamma counting expressed as standardized uptake value (SUV). (**B**) Quantification of *in vivo* PET/CT tracer uptake in myocardium across all groups and comparison of myocardial uptake of tracers in advance disease group, at 40–60 minutes post-injection, expressed as standardized uptake value (SUV). (**C**) Quantification of *ex vivo* autoradiography data expressed as photostimulated luminescence per square millimeter (PSL/mm^2^) normalized with the injected radioactivity dose and body mass, and corrected for decay of radioactivity. For all panels, values are presented as the mean ± SD (n = 4 mice per group), except in panel C, prelesion group with ^68^Ga-FOL for plaque (n = 3 mice). *P* values were calculated using a two-tailed unpaired Student’s t test.


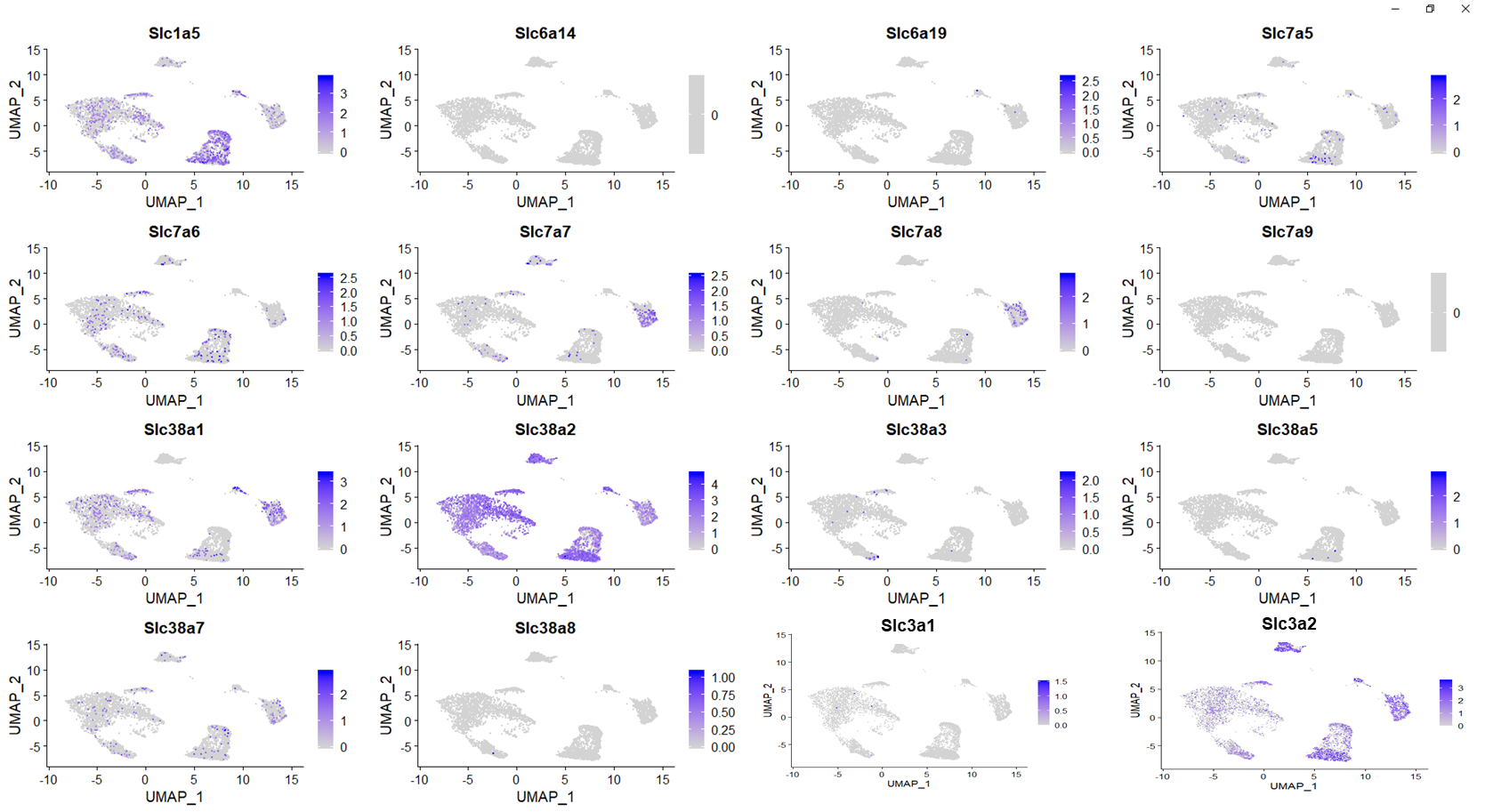


**Supplementary Figure 15.** UMAP plots showing the expression of all glutamine transporters in the 9 major cell type clusters as described in Örd *et al.*, 2023 (PMID 37060905). For cell type annotations, see Supplementary Figure 11A.


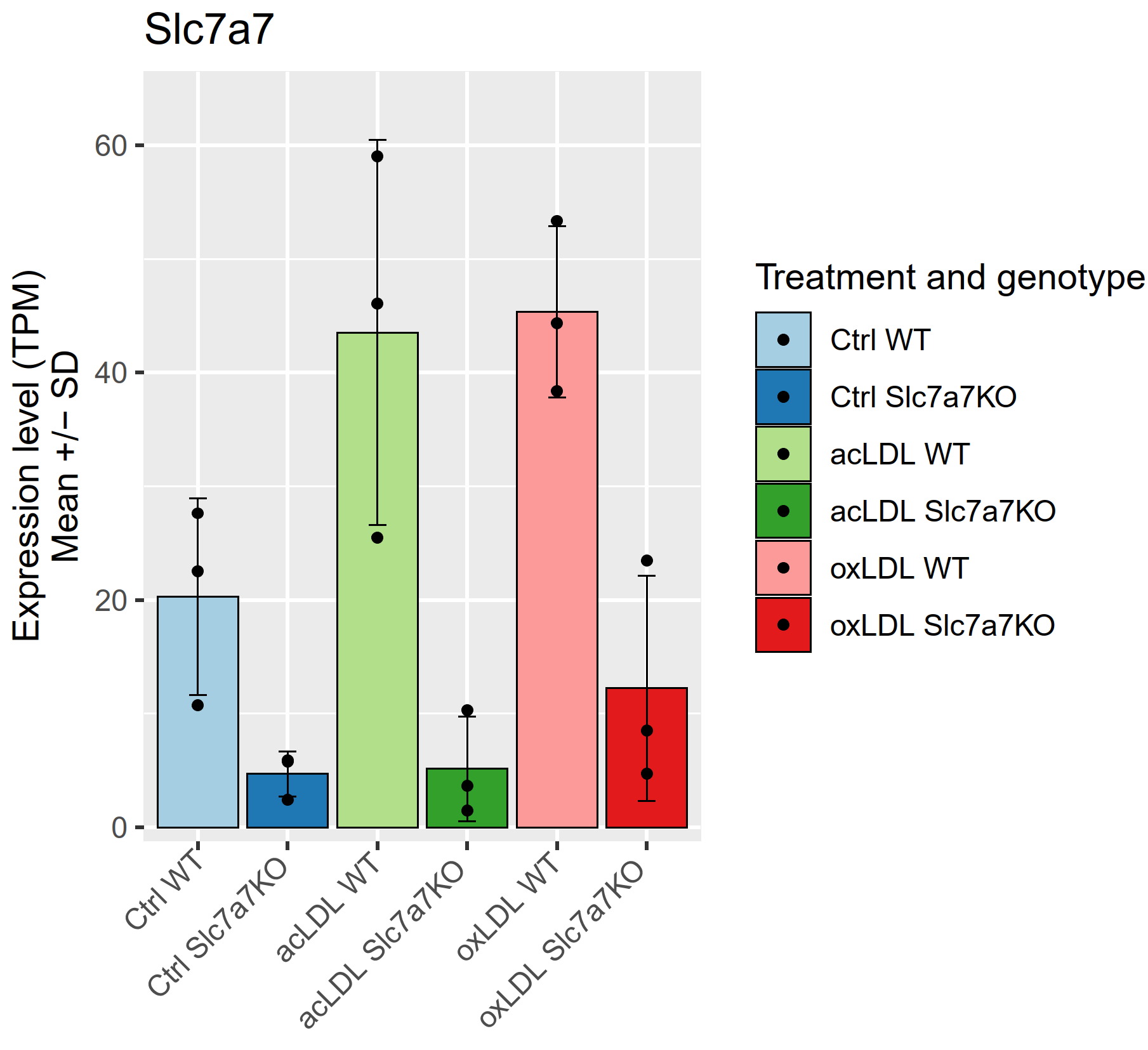


**Supplementary Figure 16.** Gene expression of *Slc7a7* in wild type (WT) and Slc7a7^LysM-/-^ knockout (KO) mouse bone marrow-derived macrophages. Cells were treated with acetylated or oxidized LDL (acLDL and oxLDL, respectively), or vehicle-treated as a control (Ctrl). TPM, transcripts per million. Sample size: n = 3 mice per genotype.
